# Design and implementation of a portable, high-speed, wide-bandwidth FPGA-based electrical impedance tomography system

**DOI:** 10.1016/j.ohx.2026.e00832

**Published:** 2026-08-22

**Authors:** Kendall R. Farnham, Ethan K. Murphy, Ryan J. Halter

**Affiliations:** Dartmouth College, 15 Thayer Drive, Hanover, NH 03755, USA

**Keywords:** Electrical impedance tomography, FPGA, Data acquisition system

## Abstract

This paper describes the design of a small form-factor, low-cost, portable electrical impedance tomography (EIT) system with a custom analog front end and field-programmable gate array (FPGA) controller. Detailed analog and digital design choices are presented and discussed in the context of currently available state-of-the art hardware components and in relation to EIT-driven system requirements. The system is capable of acquisition speeds required for real-time imaging under appropriate acquisition configurations, provides high-SNR measurements over the 100 Hz to 1 MHz target bandwidth, and has customizable acquisition parameters to allow users to optimize the system for a wide variety of imaging applications. Hardware characterization results demonstrate competitive performance relative to existing small form-factor EIT systems, while multi-frequency imaging results demonstrate successful EIT image reconstruction across the evaluated frequency range. The development of this highly configurable, high-performing, portable EIT system provides a significant step toward expanding the practical use of multi-application, real-time EIT imaging.

## Graphical abstract

**Figure:**
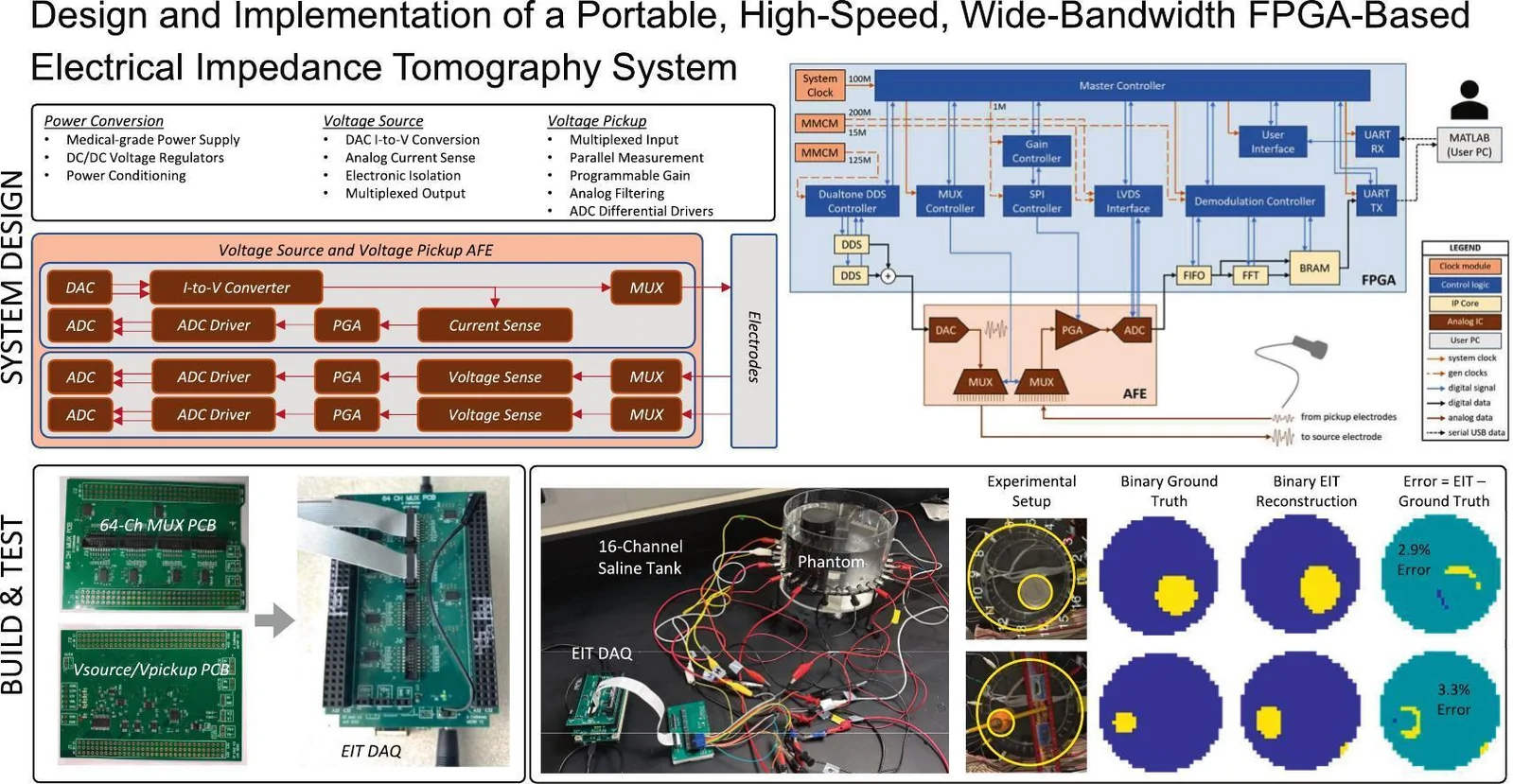

## 1 Specifications table

**Table:** 

Hardware name	Dartmouth EIT Edge-64
Subject area	Medical imaging
Hardware type	Imaging tools
Closest commercial analog	Sciospec EIT 64
Open source license	CERN-OHL-W-2.0 (hardware), MIT License (software, VHDL), CC BY 4.0 (documentation)
Cost of hardware	$650
Source file repository	https://doi.org/10.5281/zenodo.21359298

## 2 Hardware in context

Small form-factor electrical impedance tomography (EIT) data acquisition (DAQ) hardware is a low-cost and highly portable medical imaging solution in comparison to traditional modalities (e.g., ultrasound, magnetic resonance imaging (MRI), X-ray, computed tomography (CT)), making it an attractive option for providing imaging services in resource-limited areas, such as spacecraft or remote medical clinics. The US National Aeronautics and Space Administration (NASA) is particularly interested in the development of a “non-ionizing, full-body, dynamic, 3D imaging” system that can integrate with other diagnostic modalities like ultrasound [1]. A potential solution is EIT, a non-ionizing, non-radiating, non-invasive imaging technique that can be adapted for a wide range of applications, including integration with other imaging modalities. Compared with CT, MRI, and ultrasound, EIT provides lower spatial resolution but offers high temporal resolution, low cost, portability, and the ability to perform continuous, radiation-free imaging, making it particularly well suited for bedside monitoring and resource-constrained environments. EIT produces images of tissue electrical properties and has shown the potential to detect a wide range of pathologies and biological phenomena, including tissue injury, tumor malignancy, muscle atrophy, pulmonary dysfunction, and cell growth [2], [3], [4], [5], [6]. Biological tissues exhibit distinct frequency-dependent impedance behavior over the β-dispersion range (Hz to MHz), enabling EIT to discriminate between healthy and diseased tissues and monitor functional changes, such as respiration or active internal bleeding. By designing EIT hardware to acquire impedance data over a wide frequency range, we can provide imaging capabilities across a diverse range of medical applications [7].

Tissue electrical properties can be measured between multiple sets of electrodes distributed over or around an anatomic location of interest. For example, electrodes placed around the patient’s abdomen can be used to acquire complex impedance measurements relevant to the abdominal cavity structures and pathologies. In a typical EIT system, impedance data is obtained by driving a known current (I) through two electrodes and measuring the imposed voltages (V) on other electrode pairs [8]. The complex impedance (Z) is calculated using Ohm’s Law (V = IZ) for each set of current-drive/voltage-measurement pairs. This boundary impedance data is used to reconstruct images of the tissue electrical properties by solving the EIT inverse problem [8], [9], [10].

**Fig. 1:**
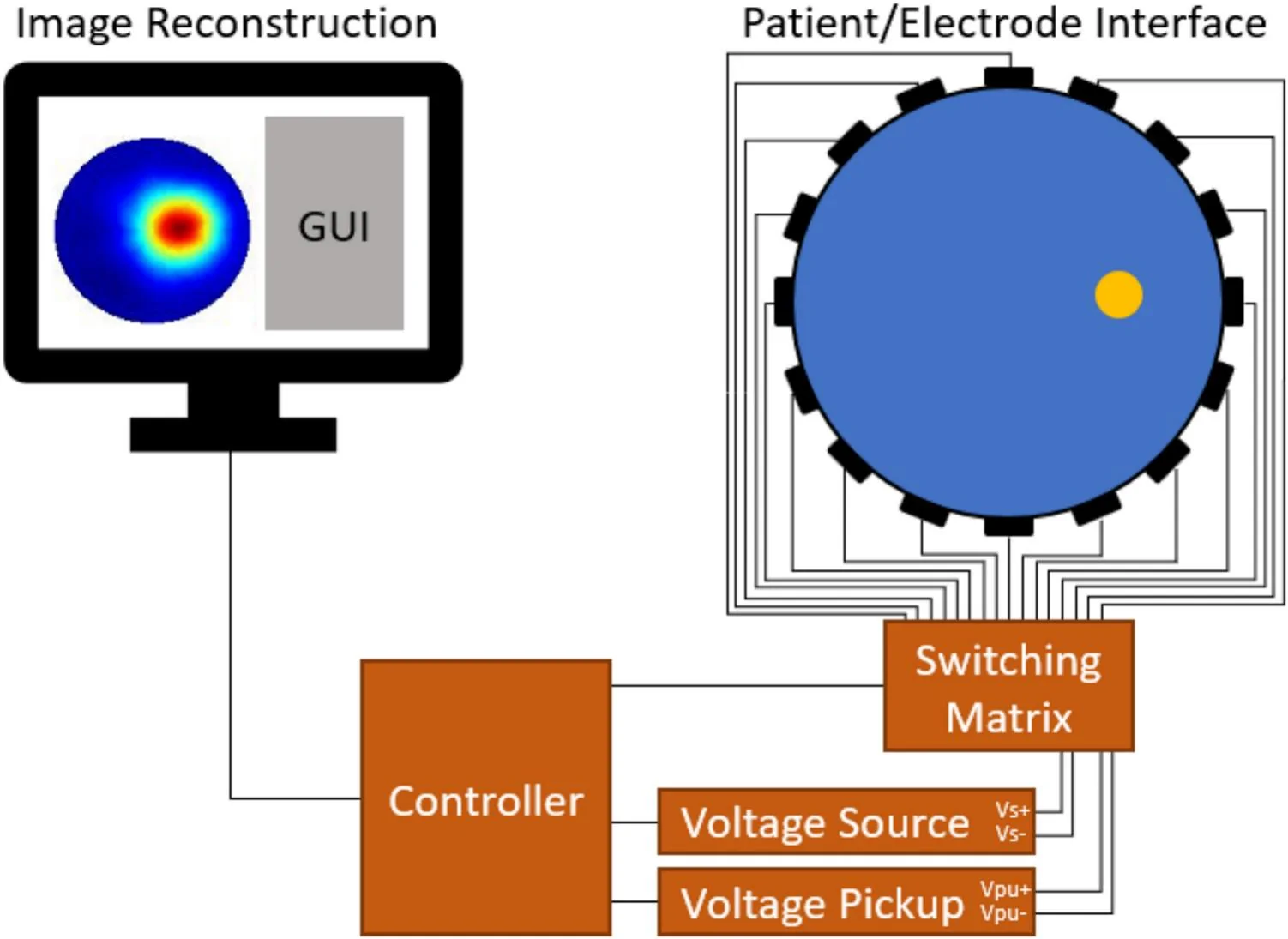
The main components of an EIT system.

An EIT system consists of an electrode array (patient interface), analog front end (AFE), controller, and software for image reconstruction [8], [11] (Fig. 1). The AFE must include a voltage or current source, a voltage pickup circuit, and a switching scheme to configure electrodes for sourcing or sensing [12]. The controller manages control of the components on the AFE and is responsible for signal filtering, demodulation, gain control, electrode switching, and communication with the user or display. User interface (UI) software is responsible for setting acquisition parameters, triggering measurements, receiving and parsing data from the controller, and extracting impedances for image reconstruction. These impedance data can be used as inputs to an EIT reconstruction software package, such as EIDORS or pyEIT, to produce the resulting EIT images [13], [14].

Over the past few decades, several high-performing large-scale EIT systems have been developed for commercial use [15], [16], but recent advancements in miniaturized integrated circuit (IC) implementation and high-precision manufacturing have shifted the focus to developing smaller, more compact hardware [17], [18]. Small form-factor system design is challenging as it requires resource sharing to reduce hardware size, which can result in a lower number of supported channels and slower acquisition speeds as resources are switched. Maximum acquisition speed is typically achieved using parallel structures, in which a separate measurement circuit interfaces each electrode channel, requiring a large AFE and subsequent digital input/output (DIO) ports to control the ICs on the parallel circuits. The limited selection of commercially available small-footprint ICs constrains small form-factor design further, as many high-performing components have large footprints or high power requirements.

Application-specific integrated circuits (ASICs) offer a potential solution for minimizing hardware size (e.g., a 4-channel EIT AFE has been implemented on a 12 × 12 mm^2^ ASIC by Rao et al. [17], and a single-channel active electrode ASIC sized 3.1 × 1.25 mm^2^ has been developed by Wu et al. [18]), but ASICs are expensive to fabricate, have lengthy development times, and are impossible to modify post-manufacturing. Conversely, field-programmable gate arrays (FPGAs) offer a highly adaptable and cost-effective hardware solution, as they can be easily reprogrammed to support updated functionality and provide a framework for rapid prototyping. FPGA-based designs can enable high performance on small form-factor hardware; for example, the KHU Mark2.5 is a portable FPGA-based system approaching the performance of large-scale systems, with an 80 dB signal-to-noise ratio (SNR) and a frequency range of 50 Hz to 500 kHz [19]. They also offer customizable functionality, such as acquiring multiple frequencies simultaneously, which can be used for real-time frequency-difference EIT (FD-EIT) imaging [20]. More recent FPGA-based EIT research has continued to demonstrate the utility of FPGA hardware for high-speed data acquisition, image reconstruction, and application-specific EIT systems across a range of biomedical and sensing applications [21], [22]. While these studies highlight the versatility of FPGA platforms, comparatively few focus on the design of open-source, compact, multi-frequency EIT hardware with high channel scalability.

Small form-factor EIT system design considerations that have not yet been addressed include:

1. Support for flexible, multi-frequency acquisition while maintaining compact hardware.
2. Open, reconfigurable hardware capable of supporting a wide range of imaging frequencies and operating conditions.
3. Compact, low-power architectures suitable for deployment in resource-constrained environments.

Flexible frequency selection allows the user to adjust the DAQ according to a specific imaging need, providing capabilities over a wider range of applications, as needed for a space-deployable EIT system. However, this flexibility is challenging to achieve since it generally requires more circuitry; typically, smaller systems will offer a limited selection of acquisition frequencies to reduce hardware size and complexity, while larger systems may be able to support this feature. FPGAs offer an ideal solution as they have the speed needed for accurate, real-time EIT imaging and the design flexibility to easily expand or customize functionality. Furthermore, FPGAs can be commercially radiation-hardened, making them a reliable platform choice for a space-deployable system.

To address these gaps, a 64-channel FPGA-based semi-parallel EIT DAQ was developed with a tunable, multi-frequency voltage source. In alignment with NASA’s Technology Roadmap [1], the DAQ supports the development of a dynamic imaging system that can integrate with other diagnostic modalities. EIT was selected for its portability, safety, high contrast between tissue types, and ability to be integrated with other modalities. This work presents the detailed design of the DAQ, with an emphasis on enabling high-performance, flexible, multi-application use in resource-constrained environments as a ground-based proof-of-concept prototype targeting future aerospace applications.

## 3 Hardware description

### 3.1 System overview

A 64-channel FPGA-based DAQ was designed as a ground-based proof-of-concept for a compact EIT imaging platform intended for future aerospace applications. Specifically, key features needed for space compliance have been outlined as:

• Small form factor and low mass to fit within constrained spacecraft environments.
• Low-resource operation, including low power consumption and minimal consumables.
• Design guided by NASA electromagnetic interference (EMI) and electromagnetic compatibility (EMC) standards [23] and International Electrotechnical Commission (IEC) 60601-1 medical safety standards [24], with the objectives of minimizing EMI and protecting the patient.
• Integration capability with existing spacecraft power systems and onboard computing.
• Multi-functionality, enabling the DAQ to support multiple plug-and-play probes for diverse physiological applications.
• Radiation-tolerant and reconfigurable hardware, ensuring mission resilience and post-deployment adaptability.
• Autonomous and user-friendly operation, allowing non-specialist crew to perform imaging without telemedical support.

To satisfy medical safety standards and support future aerospace applications, the proof-of-concept DAQ was developed to meet the design objectives listed in Table 1. The system implements high-speed, wide-bandwidth multi-frequency impedance acquisition with compact, low-power circuitry. The DAQ integrates:

1. A medically compliant power supply (IEC 60601-1).
2. An Artix-7 FPGA (Xilinx), chosen for its low cost and low power consumption. Although the Artix-7 FPGA is not radiation hardened, the FPGA design is portable to radiation-tolerant or radiation-hardened FPGA families for future aerospace implementations.
3. A custom AFE incorporating a multi-frequency, multi-tone voltage source and dual-channel voltage pickup circuits.
4. A MATLAB-based graphical user interface (GUI) for intuitive user control, parameter selection, and visualization of real-time impedance data.

**Table 1:** EIT system requirements.

Specification	Target	Justification
Frame rate	>30 fps	Capture fast-moving processes, e.g., bleeding, cardiac function
Impedance precision	1 Ω	For more accurate EIT reconstruction
Impedance range	10 Ω–3 kΩ 1 pF–30 nF	Flexibility for multi-application use: typical human body impedance range is 30 – 1500 Ω and 2 pF – 22 nF [25]
Bandwidth	100 Hz–1 MHz	High sensitivity to bioimpedance, limited by performance of existing ICs
No. channels	64	Flexibility for multiple applications
SNR	80 dB	For more accurate EIT reconstruction (>60 dB required for EIT reconstruction [26], 80 dB to compete with existing high-performance systems)
Max current	<10 mA	IEC 60601-1 safety compliance
Power supply	Wall power	IEC 60601-1 compliance, minimize power draw
Size	<12 × 12 ×12 cm	Minimize volume
Mass	<1 kg	Minimize mass
User interface	MATLAB GUI	Single-program interface for ease of use, MATLAB software required for EIT reconstruction

**Fig. 2:**
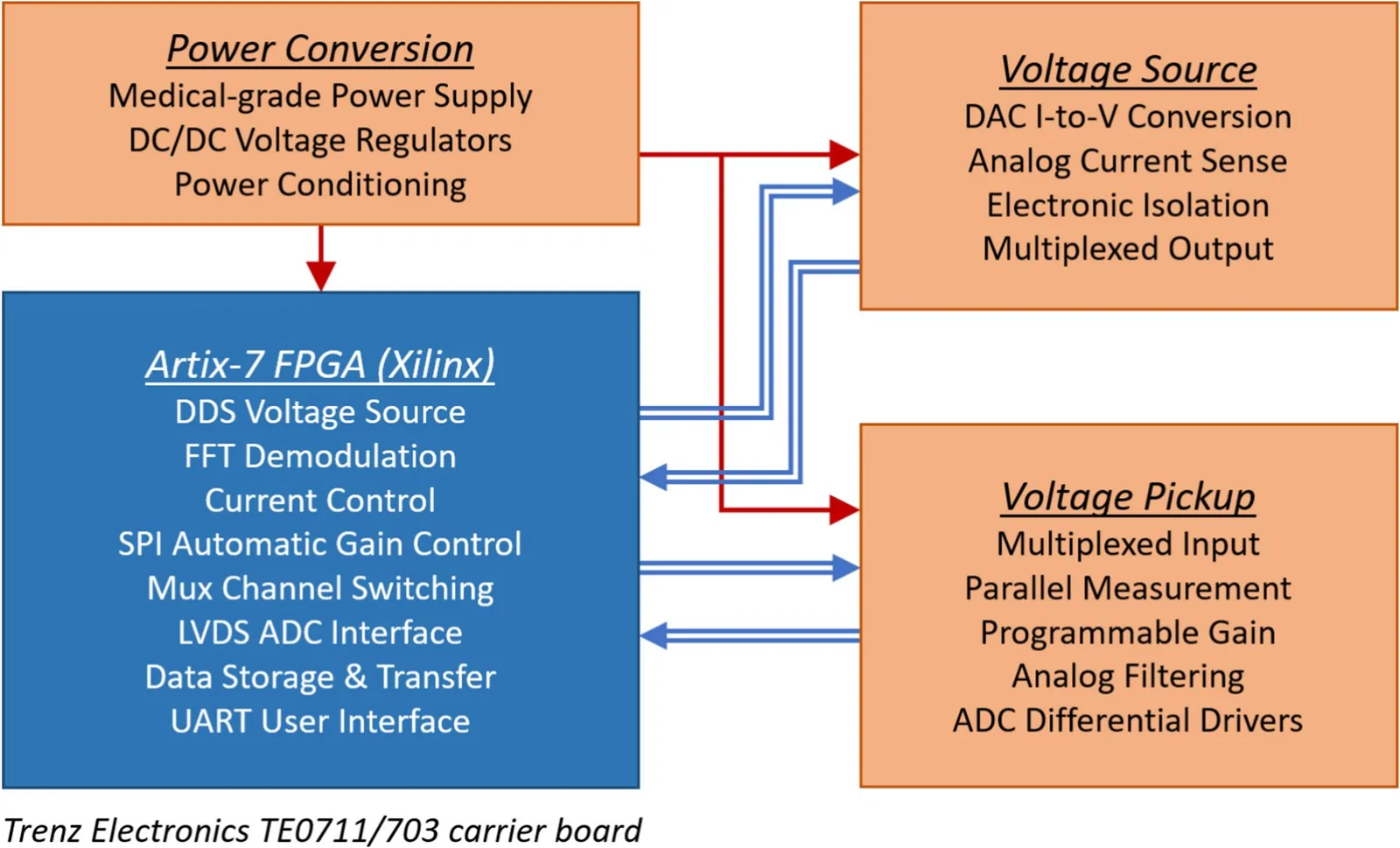
High-level block diagram of the EIT DAQ functions. An Artix-7 FPGA is used for the digital controller (blue), and the custom AFE includes the power management, voltage source, and voltage pickup circuits (orange). Blue arrows indicate data signals, and red arrows indicate power lines. (For interpretation of the references to colour in this figure legend, the reader is referred to the web version of this article.)

The high-level block diagram in Fig. 2 outlines the primary functionality of the DAQ. The Artix-7 FPGA was chosen for its low cost and design flexibility, as compared to an analog implementation, as well as its low power requirements and high-speed capabilities. Commercially available Trenz Electronic FPGA carrier boards (TE0703-06 and TE0711-01) were used as the interface to the Artix-7 FPGA. The AFE sits on top of the TE0703 DIO pins and implements a wide-bandwidth, dual-channel measurement scheme that enables multi-frequency impedance acquisition while maintaining high SNR (>80 dB) and precision (≤1 Ω). The 64-channel interface provides flexibility for multi-electrode configurations, allowing the same system to be used for different imaging tasks such as pulmonary monitoring, muscle assessment, or targeted diagnostic scans.

For the voltage source portion of the circuit, a single- or multi-tone direct digital synthesis (DDS) signal is generated on the FPGA, converted to a voltage using a digital-to-analog converter (DAC) and custom differential-to-single-ended current-to-voltage (I-to-V) circuit, and sent to the current sensing and patient isolation circuits. The sensed current on the voltage source is amplified and buffered, then converted to the digital domain using a custom differential driver circuit interfacing an analog-to-digital converter (ADC). The ADC digital output is sent to the FPGA for demodulation and gain control to ensure injected current amplitude is within patient safety limits (IEC 60601-1). Capacitors located after the multiplexers (MUXs) at the electrode interface isolate the patient from direct current (DC) during injection and provide alternating current (AC) coupling for the voltage sense circuit.

A similar circuit topology is used for voltage pickup, in which the sensed voltage is amplified, buffered, and sent to an ADC driver circuit to interface the measured signal to the ADC and FPGA for demodulation. Two identical voltage pickup circuits are implemented in parallel to increase acquisition speed for imaging applications requiring a high frame rate – with both voltage pickup channels acquiring data from different electrodes simultaneously, acquisition time can be cut in half.

Commands are sent from a laptop to the FPGA via universal asynchronous receiver-transmitter (UART) communication to set system parameters and run the acquisition. A UART controller on the FPGA receives these commands and sends impedance data back to the laptop for parsing and data visualization. A graphical user interface (GUI) developed in MATLAB allows the user to easily set acquisition parameters, run the DAQ, and view the resulting data or reconstructed image.

**Fig. 3:**
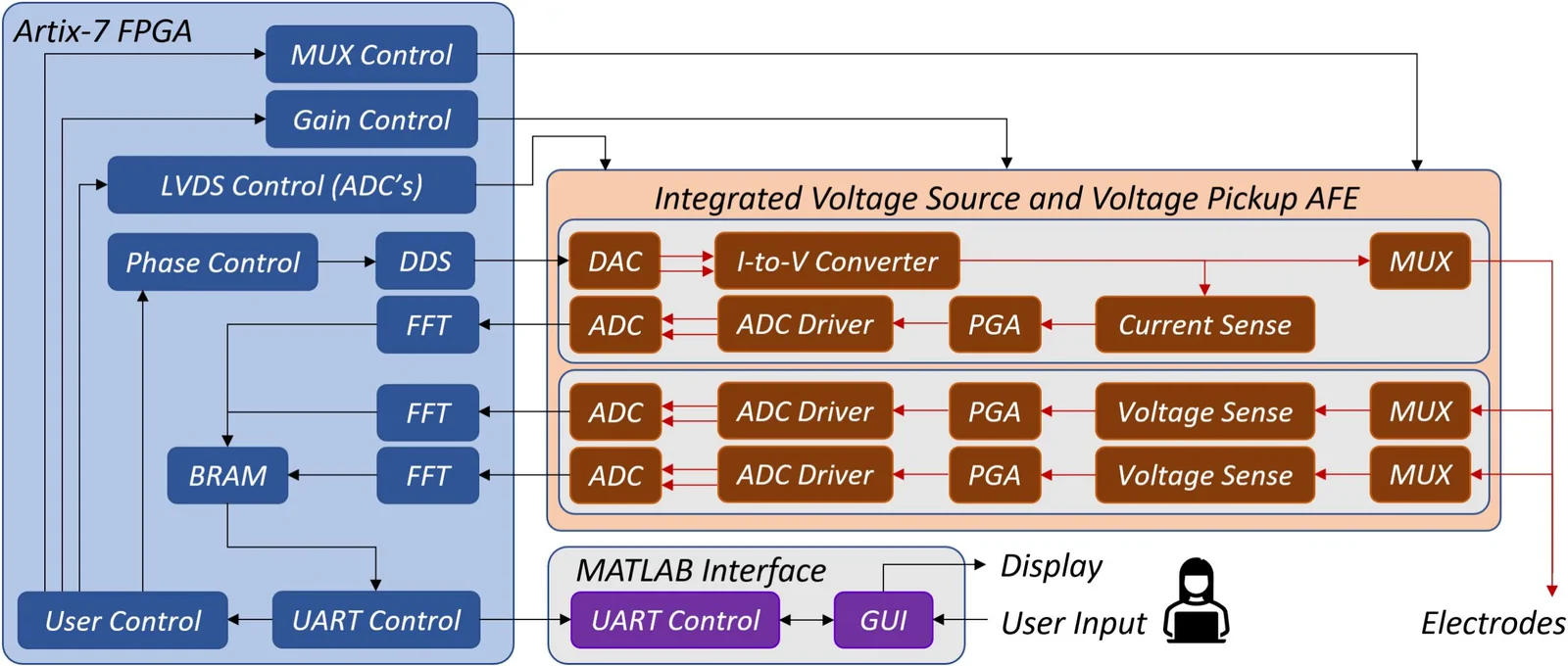
Detailed block diagram of key functionality on the EIT DAQ. Digital blocks implemented on the FPGA are shown in blue, and analog circuits are in orange. Signal paths are denoted with arrows and represent digital (black) and analog (red) signals. A MATLAB user interface provides a user-friendly tool to set DAQ parameters, trigger acquisition, and view results. (For interpretation of the references to colour in this figure legend, the reader is referred to the web version of this article.)

**Fig. 4:**
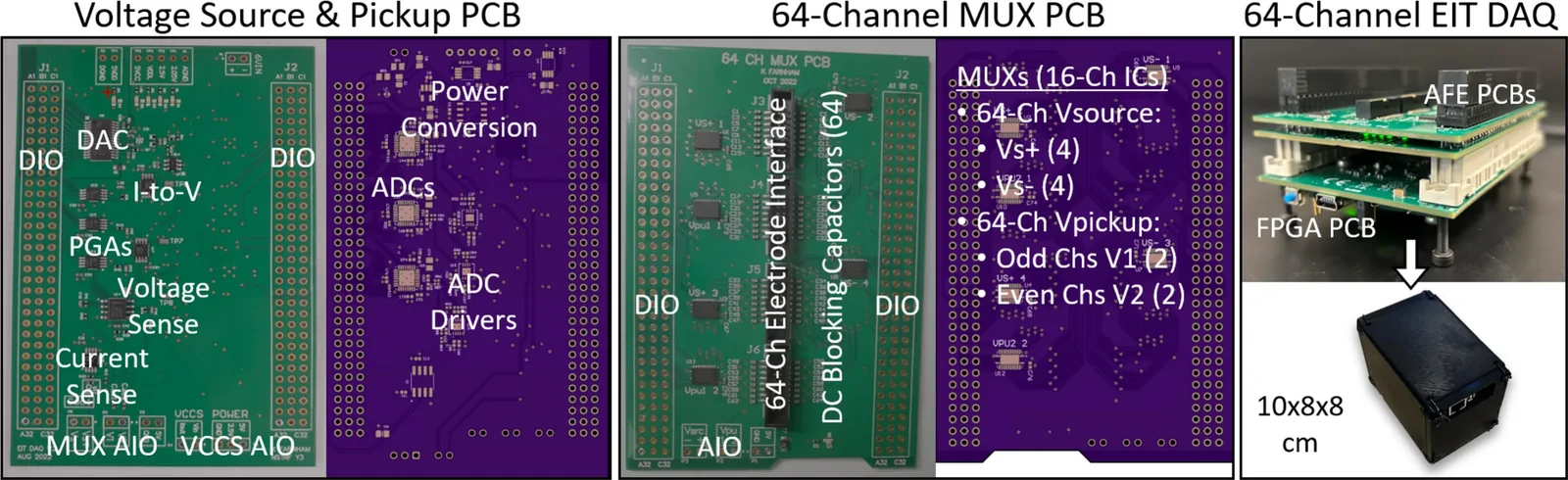
Integrated FPGA-based 64-channel EIT DAQ. The custom AFE comprises of the integrated voltage source and pickup PCB and a 64-channel MUX PCB, which stack on top of an Artix-7 (Xilinx) FPGA carrier board (Trenz Electronic, TE0703-06 & TE0711-01) via 96-pin connectors for a full system size of 10x8x8 cm. A 3D-printed case was designed to house the DAQ to protect the hardware for reliable use.

The analog circuits in Fig. 3 were integrated onto a small form-factor 64-channel AFE and designed to stack directly on top of the TE0703-06 via 96-pin connectors, resulting in a full system size of 10 × 8 × 8 cm (Fig. 4). To conserve board space and provide an adaptable electrode interface, the AFE was divided into two printed circuit boards (PCBs): one PCB houses the voltage source and pickup circuits, while the other implements a 64-channel MUX interface to the electrodes. Additionally, the voltage source/pickup PCB has an extra interface option that permits it to drive a voltage-controlled current source (VCCS), offering flexibility for both voltage-driven and current-driven system designs. Power conversion for the AFE circuitry is implemented on the voltage source/pickup PCB, with these power supplies simultaneously powering the MUX PCB (and optionally a VCCS PCB) through stacked connector pins to uphold the compact design. These PCB designs and annotated circuit schematics are provided in the design files.

The long-term vision of this system is to contribute to an integrated medical imaging suite capable of self-guided injury diagnosis and physiological monitoring during long-duration missions beyond low-Earth orbit. More broadly, the system has features that can be tailored for a variety of use cases, specifically:

• Portable, multi-frequency EIT acquisition (100 Hz–1 MHz) with high SNR enables spectroscopy-style studies (β-dispersion) and time/frequency-difference EIT on benchtop phantoms, animal models, or human subjects, with >80 dB SNR (with averaging) and ≤1 Ω precision for reproducible measurements.
• Configurable 64-channel electrode interface allows for flexible current-drive/voltage-sense patterns and electrode geometries for diverse tasks (lung/diaphragm monitoring, muscle health, edema/bleed localization, tissue characterization) without changing the core DAQ.
• Dual-channel, semi-parallel architecture reduces per-frame acquisition time, supporting higher frame rates for dynamic processes (e.g., respiration, perfusion, cardiac motion).
• MATLAB GUI and UART API facilitate automated protocols, scripted sweeps (frequency, gain, patterns), image reconstruction/analysis pipelines, and 3D co-registration with other imaging modalities.
• Open, modifiable FPGA/AFE platform allows researchers to extend the design (e.g., custom current drivers, alternate MUX topologies, radiation-tolerant parts) for novel probes, field deployments, or education/training labs.

The following sections describe the implementation details of each functional block shown in Fig. 3. Section 2.2 focuses on the analog circuits, including the power management, voltage source, measurement, and multiplexing circuits, while Section 2.3 describes the FPGA implementation, including the digital signal processing architecture, timing analysis, and MATLAB software interface.

### 3.2 Analog front end

A custom AFE was designed to implement the multi-channel voltage source, voltage pickup, and power management functionality. The AFE PCBs utilize a 6-layer board stack, with internal layers used for ground and power planes and outer layers used for signal traces. The 64-channel MUX PCB was designed to stack on top of the voltage source and pickup PCB, which interfaces to the FPGA board via two 96-pin connectors, resulting in a compact full system design. The area footprint of the AFE is 10x6.5 cm, with height determined by connector header heights and pin lengths.

#### 3.2.1 Power conversion

A medically compliant wall-mount 5 V power supply (Model ME30A0503B01) powers the DAQ via a port on the TE0703-06. The medical supply is rated for 5 V and 4.0 A, providing 20 W of wall-driven power that is distributed through stacked connectors to both the TE0703-06 and the AFE for conversion (power conversion on the TE0703-06 is independent of the circuit implemented on the AFE). DC/DC converters generate regulated supply voltages for the analog and digital components to prevent damage to sensitive ICs. Separate digital and analog supply voltages, power planes, and ground planes were implemented in the PCB design to reduce noise and decouple circuits.

Low drop-out (LDO) voltage regulators provide the analog and digital 5 V power supply rails (TPS77350, Texas Instruments), and a 2.5 V LDO regulator (AP2139, Diodes Inc.) drives the common mode supply pins on the ADCs. Ultra-high-precision (0.02% tolerance), ultra-low-noise voltage reference ICs (ISL21090, Renesas, and MAX6126, Maxim Integrated) drive the common mode reference voltages on the current sense and ADC driver circuits, ensuring precise/equivalent voltage level shifting of the AFE signals used for computing complex impedances on the FPGA. Decoupling capacitor values, size, and placement for each IC were chosen based on datasheet recommendations.

The 5 V LDO regulator has a maximum output power of 1.25 W. The AFE power consumption can be estimated using the maximum supply current specifications from the IC datasheets and is dominated by the DAC, three ADCs, and twelve MUX ICs. Under these worst-case assumptions, the total AFE power consumption is approximately 0.76 W, well below the regulator’s 1.25 W limit, and consistent with the low power requirements for future space-based EIT applications.

#### 3.2.2 Voltage source and current sense

A 125 MSPS 14-bit DAC (THS5671A, Texas Instruments) is used to convert the single- or multi-tone digital DDS signal to differential currents, and a differential-to-single-ended current-to-voltage circuit converts the DAC outputs to a voltage for the voltage source. Although it is possible to use the DAC’s differential current outputs directly as a current source for the EIT DAQ, this approach poses significant design challenges, particularly concerning output impedance. The THS5671A DAC provides differential output currents ranging from 2-20 mA set by an external bias resistor, with a typical output impedance of 300 kΩ and 5 pF at full scale. However, for EIT applications, a much higher output impedance (>1 MΩ) is needed to achieve the required SNR for accurate EIT reconstruction (>60 dB), and a low output capacitance (<5 pF) is desired to reduce current noise for maximizing bandwidth and impedance range [26], [27]. Consequently, increasing the output impedance of the current driver and ensuring stability across the target bandwidth would require significant compensation circuitry to be designed, unnecessarily complicating the analog design.

**Fig. 5:**
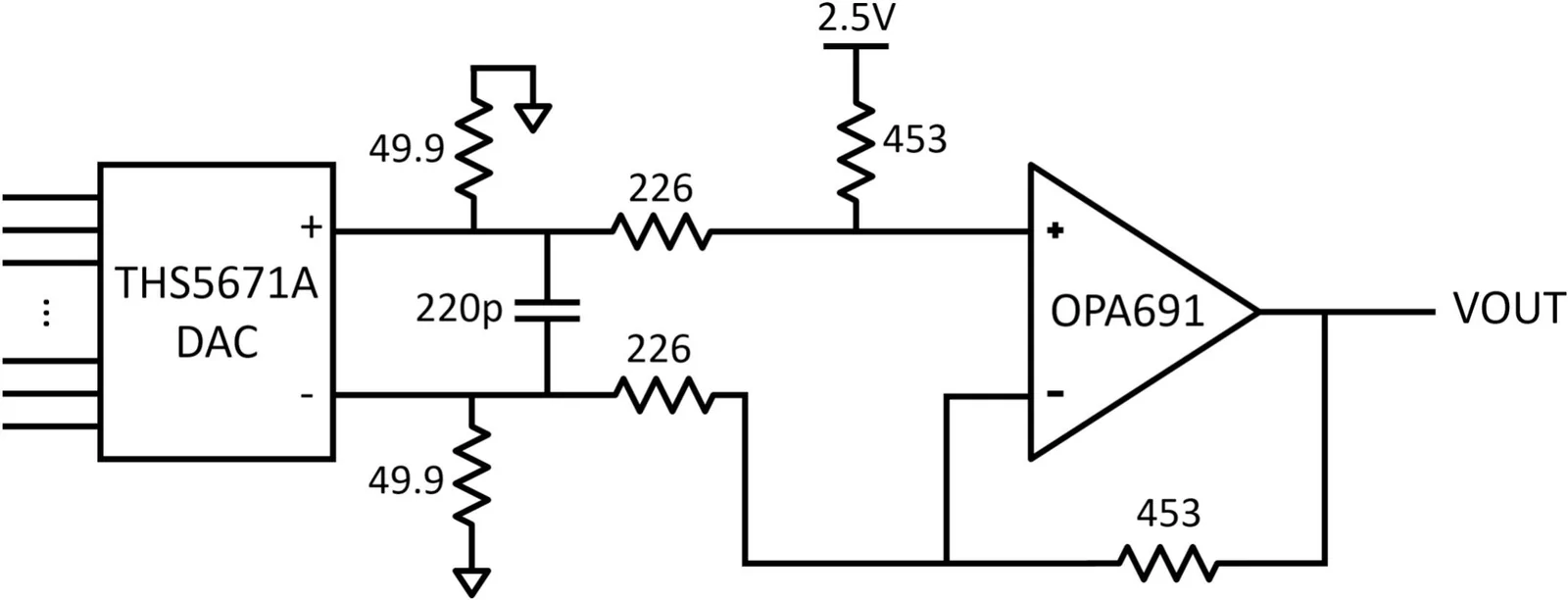
DAC differential-to-single-ended current-to-voltage conversion circuit used to generate the voltage .

Source. This circuit converts 20 mA differential currents from the DAC to a 4 Vpp single-ended voltage (VOUT) and uses a high-precision voltage reference IC to level-shift the signal up to 2.5 V

Additionally, patient safety is a critical feature of an EIT system, and thus monitoring the injected current to ensure compliance with IEC 60601-1 safety is standard practice. This is simple on a single-ended source, as only a single sense resistor and measurement circuit is needed. Differential signals, on the other hand, require precisely matched sense resistors on each output to avoid signal distortion, resulting in a more complicated design and tighter component specifications. To protect the patient from harmful DC signals, capacitors are typically placed after the source at the electrode interface. Capacitors on the current drive output can saturate the signal at lower frequencies due to charging, thereby limiting the effective bandwidth of the EIT system [26]. Given the design complexities associated with differential topologies and current sources, a single-ended voltage source was chosen for the EIT DAQ implementation, and subsequently a differential-to-single-ended current-to-voltage circuit was designed for the DAC interface (Fig. 5).

**Fig. 6:**
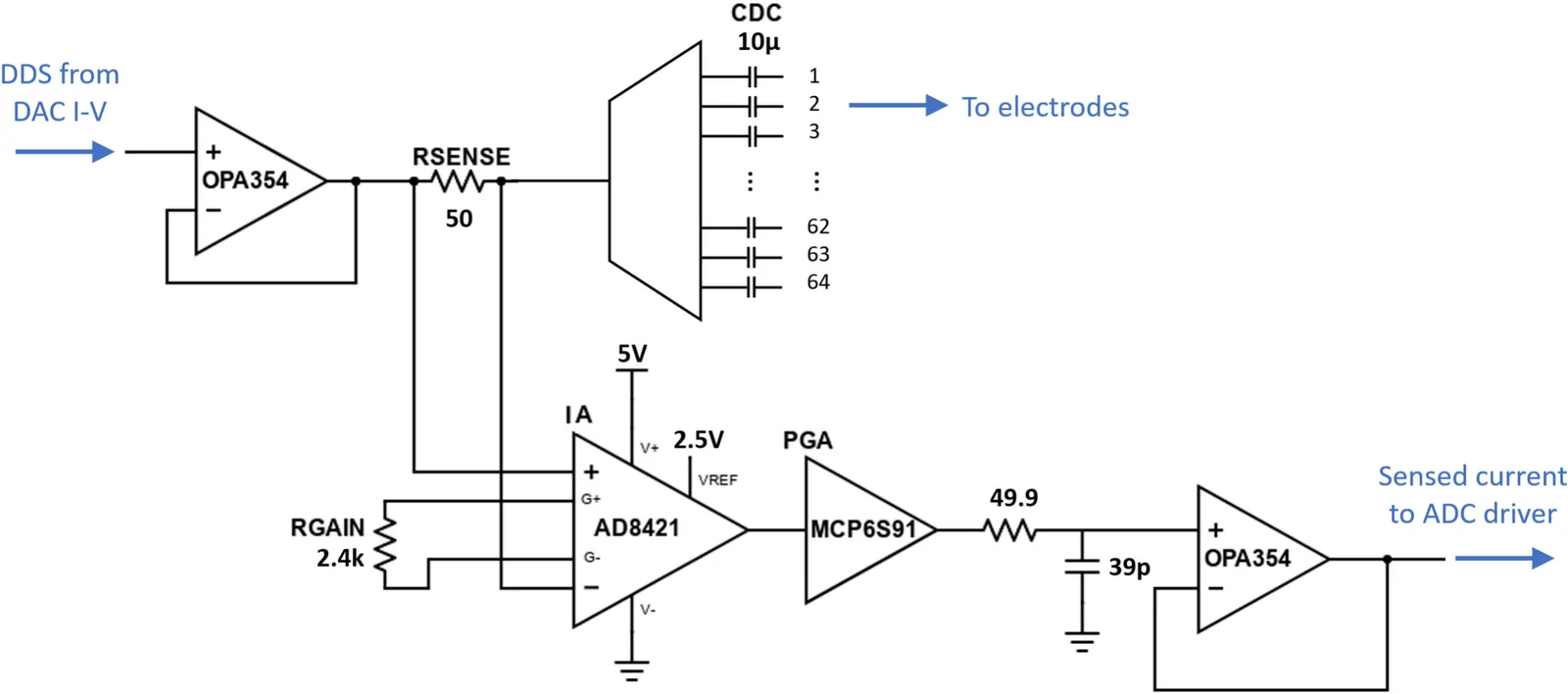
A current sense circuit measures the injected load current from the voltage .

Source. Current is measured across a sense resistor (R_sense_) using an IA, and DC blocking capacitors (C_DC_) are placed on the electrode interface to block DC from entering the patient (required by IEC 60601-1)

The current sense circuit in Fig. 6 senses the source current injected into the patient by measuring the voltage across a sense resistor (R_sense_) using an AD8421 (Analog Devices) instrumentation amplifier (IA). IAs specialize in amplifying very small signals in the presence of high noise, making these op-amps extremely useful for measuring a small voltage difference across the known R_sense_ load to calculate current. Additionally, IAs have very high input impedances, which ensures the current measured across the sense resistor is the same amplitude as the current passing through the patient-facing electrodes; accurate detection of this current is crucial for image reconstruction and ensuring patient safety. The AD8421 is currently the widest bandwidth IA that runs on a 5 V single supply, with a 10 MHz bandwidth, 35 V/μs slew rate, 0.01% differential gain error, 30 GΩ input impedance, and 80 dB common-mode-rejection-ratio (CMRR) at 20 kHz. The IA has the lowest bandwidth of any component on the AFE and therefore limits the system bandwidth when driving higher-impedance loads (>1 kΩ). For applications requiring accurate broadband measurements on higher-impedance loads, adding a VCCS, such as a Howland current source, to the VCCS AIO connector (Fig. 4) is recommended to maintain measurement bandwidth (VCCS implementation details are described on page 1 of *EIT-AFE-Schematics.pdf*).

The value of R_sense_ was determined by the bandwidth, slew rate, expected loads, and voltage range requirements. A DC blocking capacitor (C_DC_) is needed on the electrode interface to ensure no DC current flows through the patient, which means R_sense_, C_DC_, and the patient load are all in series, creating a voltage divider as well as a high-pass filter at the patient interface. Since this is a voltage source, maximizing the possible output voltage amplitude is preferred in order to have the highest SNR; ideally, this would be achieved with a sense resistor value significantly lower than the load impedance. However, we also need to ensure the corner frequency of the high-pass filter created by R_sense_ and C_DC_ is sufficiently low to meet the low-frequency bandwidth target of 100 Hz. A smaller R_sense_ value would yield a larger output voltage on the voltage source electrode, but the signal measured by the IA would be small and a large C_DC_ would be required to meet the 100 Hz target.

For the 64-channel AFE implementation, a 50 Ω R_sense_ and sixty-four 10 μF X5R C_DC_s were chosen (10 μF was the largest value capacitor available in 0402 chip size), and a 2.4 kΩ gain resistor was used on the IA for a +5.125 V/V signal gain. The addition of the MUX adds a series resistance (the MUX ICs have a typical on-resistance of 90 Ω), which results in a high-pass 3 dB cutoff frequency of approximately 113 Hz, causing minimal disruption to low-frequency performance. Given this configuration, a 10 mA source (the maximum allowable current for frequencies ≥100 kHz) yields a 500 mVpp signal across R_sense_ and 2.56 Vpp signal at the IA output, falling well within the AFE’s dynamic range. After the IA, the current sense signal is dynamically amplified and buffered by a programmable gain amplifier (PGA) to interface a differential ADC driver and ADC. Once this voltage signal has been demodulated on the FPGA, the load current is calculated using Ohm’s Law. If necessary, the DDS source amplitude can be adjusted digitally using the software interface to ensure that the injected current remains below the safety threshold specified in IEC 60601-1.

Ensuring a safe current injection amplitude is critical in any EIT system. Since this design leverages a voltage source, reducing the maximum possible root mean squared (RMS) current injected into the patient ($I_{\mathrm{RMS}}$) can be done by reducing the amplitude of the DAC I-V voltage (V_OUT_ in Fig. 5) or increasing the value of the load (the load impedance is the series combination of R_sense_, C_DC_, and the MUX on-resistance) on the circuit. Notes on tuning the DAC I-V circuit to a different voltage gain are available on the schematic (page 4 of *EIT-AFE-Schematics.pdf*). In the presented 64-channel AFE implementation (2 V V_OUT_, 50 Ω R_sense_, 10 μF C_DC_, and 90 Ω R_MUX_), the maximum RMS current ($I_{\mathrm{RMS}}$, A) injected into the load at 100 Hz can be approximated by (1):

(1) $$I_{\mathrm{RMS}}=\frac{V_{\mathrm{out}}/\sqrt{2}}{\left|Z_{\mathrm{load}}\right|}=\frac{2/\sqrt{2}}{\sqrt{{\left(50+90\right)}^{2}+159^{2}}}\approx 6.7mA$$

This value exceeds the safe limit (0.1 mA RMS at 100 Hz), so for applications expecting small resistive loads (<1 kΩ) and requiring low-frequency measurements, it is crucial to set the digital DDS divider to an appropriate value. The default settings divide the digital DDS signal by 16, which will yield a peak voltage of 125 mV; this results in an RMS load current of 76.8 µA for a 1 kΩ load, falling within the safe limits set by IEC 60601-1. Note that a division by 16 results in a decrease in SNR of approximately 24 dB; for applications focused on high-frequency (>100 kHz) measurements or large loads (>1 kΩ), this value should be decreased using the software interface to improve SNR.

#### 3.2.3 Dual-channel voltage pickup

**Fig. 7:**
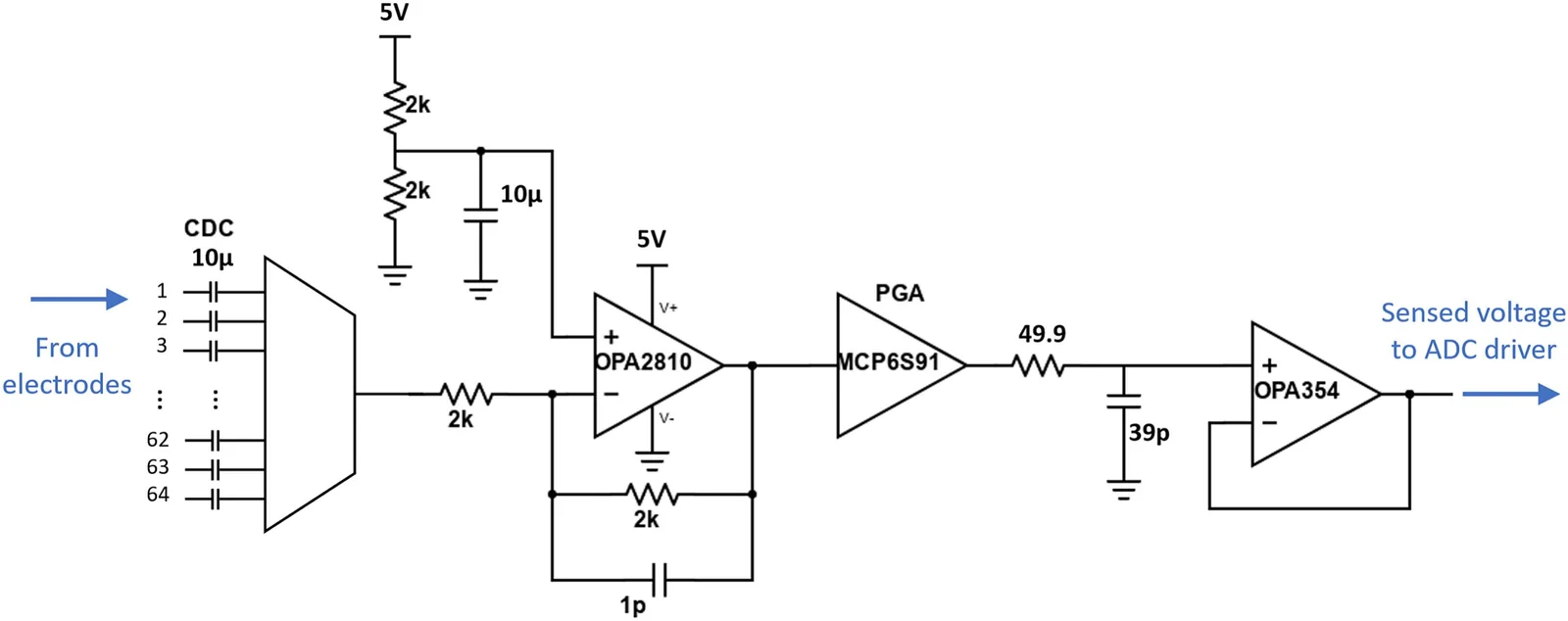
Single-channel voltage pickup circuit senses a single-ended voltage from the electrodes. The DC blocking capacitors on the electrode interface serve as AC coupling capacitors to allow the OPA2810 circuit to shift the measured signal common-mode voltage from 0 V to 2.5 V.

Two voltage pickup circuits are implemented in parallel to provide a dual-channel measurement scheme (Fig. 7). Each pickup channel senses the single-ended voltage on the electrode interface and shifts the signal up to 2.5 V using the OPA2810 (Texas Instruments) dual op-amp (one input per channel). The OPA2810 op-amp was chosen for its high CMRR (92 dB), fast slew rate (88 V/μs), high input impedance (12 GΩ), and excellent noise characteristics. An AC coupling capacitor is needed on the input of the voltage sense to isolate the patient from the electronics: for the 64-channel AFE, the DC blocking capacitor on the electrode interface serves as the AC coupling capacitor as well, which is allowable because an electrode will not be used for both source and pickup simultaneously during an impedance measurement.

**Fig. 8:**
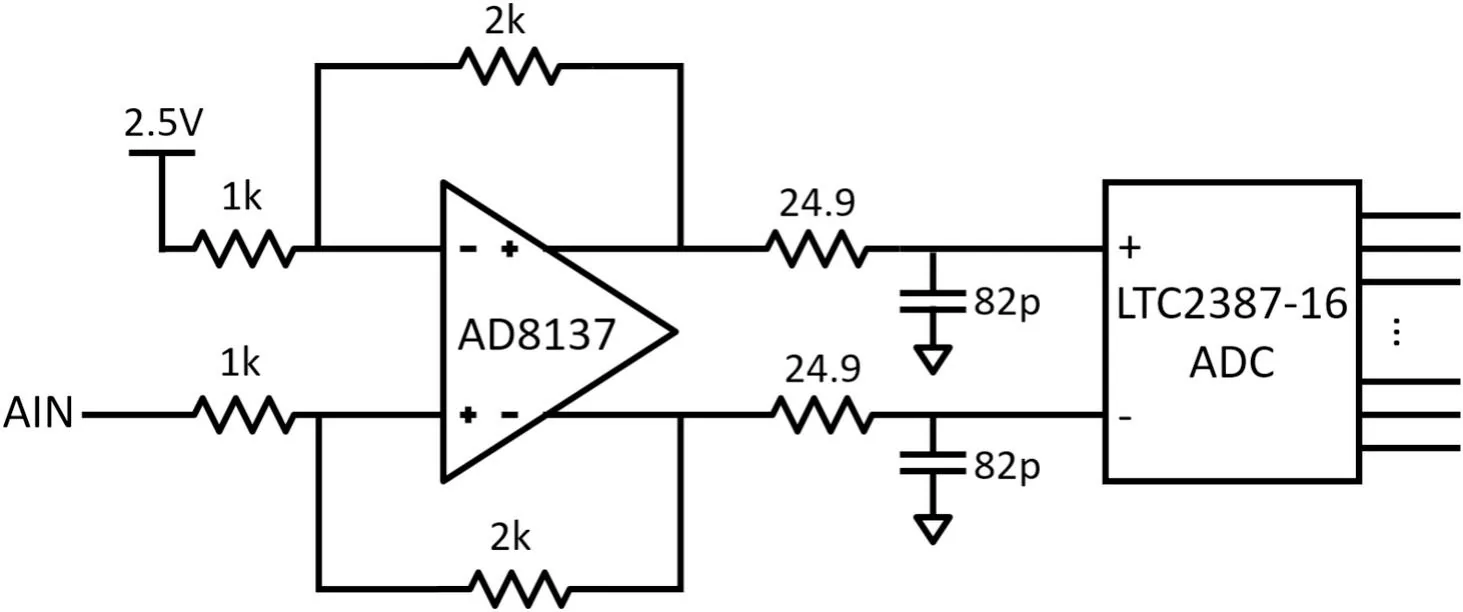
ADC differential driver circuit converts single-ended voltage signal (AIN) to level-shifted differential voltage inputs for the LTC2387-16 ADC.

Like the current sense circuit, the sensed voltage is dynamically amplified by a PGA, buffered, and sent to an ADC driver circuit to be converted to the digital domain for demodulation on the FPGA. The MCP6S91 PGA (Microchip Technology Inc) was chosen for its wide bandwidth and provides gains of 1, 2, 4, 5, 8, 10, 16 and 32 set through serial peripheral interface (SPI) commands from the FPGA. A 15 MSPS 16-bit ADC (LTC2387-16, Analog Devices) converts analog signals to the digital domain for demodulation on the FPGA, and a custom differential driver circuit drives the ADC inputs (Fig. 8).

The LTC2387-16 ADC can accept differential inputs with voltages ranging from 0 to 4.096 V, so a level-shifting ADC driver circuit was designed to convert the AFE’s single-ended voltage signals centered at 2.5 V to differential signals centered around 2.048 V. The use of an ADC driver is essential for optimizing ADC performance to achieve high SNR and signal stability, and it is particularly crucial on the current sense circuit, as the instrumentation amplifier in the current sense circuit cannot drive a capacitive load. The AD8137 (Analog Devices) low-power differential driver IC was chosen for its wide bandwidth (107 MHz), high slew rate (375 V/μs), high CMRR (90 dB), and high input resistance (800 kΩ). Bias voltages (2.5 V and 2.048 V) on the AD8137 circuit are driven by ultra-high-precision, ultra-low-noise voltage reference ICs (ISL21090 and MAX6126) to provide equivalent voltage level shifting of the sampled analog signals, allowing for more accurate and repeatable complex demodulation on the FPGA. The passive low-pass filter following the AD8137 has a 3 dB cutoff frequency of 78 MHz per datasheet guidelines for driving the LTC2387-16 ADC at a 15 MSPS sampling rate [28].

#### 3.2.4 64-channel multiplexer interface

The MUX circuit provides the switching scheme to allow any electrode to be selected as a source (Vs+), sink (Vs-), or pickup (V1/V2), creating a highly flexible multi-channel AFE. A 16-channel MUX is used to wire a single input to 1 of 16 outputs selected using DIO (enable and 4 select bits) from the FPGA, leaving the other 15 outputs open. This feature allows for each electrode to interface the voltage source MUXs (Vs+ and Vs−) and a voltage pickup MUX (V1 or V2) without signal interference. The FPGA selects one electrode as Vs+, one as Vs-, and then performs single-ended measurements on the remaining electrodes by switching through the voltage pickup MUX channels. The MUXs can be used in parallel when the enable pin is utilized, allowing the electrode channel count to be increased. To scale the 16-channel circuit to 64 channels, four cascaded MUXs are used on each voltage source output (Vs+, Vs−), and two cascaded MUXs are used on each voltage pickup input (V1, V2) to allow for high-speed, semi-parallel single-ended measurements on all 64 channels.

The MUX source and drain capacitance, as well as on-resistance and load impedance, result in a frequency-dependent transfer function. At higher frequencies, the MUX source/drain capacitance introduces attenuation and phase shifts, reducing the measured signal amplitude and consequently, the effective SNR. In testing, we found that high source and drain capacitance on the MUX can negatively impact the bandwidth when driving loads above 1 kΩ. The 16-channel 74HC4067 MUX (Nexperia) was chosen for the design as it has the lowest source/drain capacitance (45 pF) of all commercially available 16, 32, or 64-channel MUX ICs while meeting all other requirements. The 74HC4067 datasheet specifies that for a 4.5 V supply, the typical on-resistance is 90 Ω (max 160 Ω) with up to 10% difference between channels. Although this resistance is significant relative to the lowest measured loads (e.g., 10 Ω), the tetrapolar measurement configuration independently measures the injected current, so the source-path MUX resistance is largely accounted for when calculating impedance from V_load_/I_sense_. The impact of these channel-to-channel variations is reduced when using time-difference EIT reconstruction, as both the reference and measurement data are acquired through the same signal paths. In contrast, for absolute EIT imaging (without a reference dataset), channel-dependent gain and phase errors may need to be calibrated using a calibration phantom to improve accuracy.

### 3.3 Digital implementation

An FPGA controller provides the interface between the user and AFE: custom logic defines DAQ functionality, a serial interface communicates with the UI, and DIO controls components on the AFE. The Artix-7 FPGA was chosen for its low cost, design flexibility, low power requirements, and high-speed capabilities. Notably, all logic designed for the Artix-7 FPGA can be easily ported to one of Xilinx’s space-grade FPGAs, as the lowest performing space-grade FPGA has a similar architecture but with more fabric and IO than that on the Artix-7 (commercially available space-grade FPGA modules are very expensive (> $15,000), and due to budget constraints only low-cost FPGA modules (< $1,000) were considered for prototype DAQ development).

**Fig. 9:**
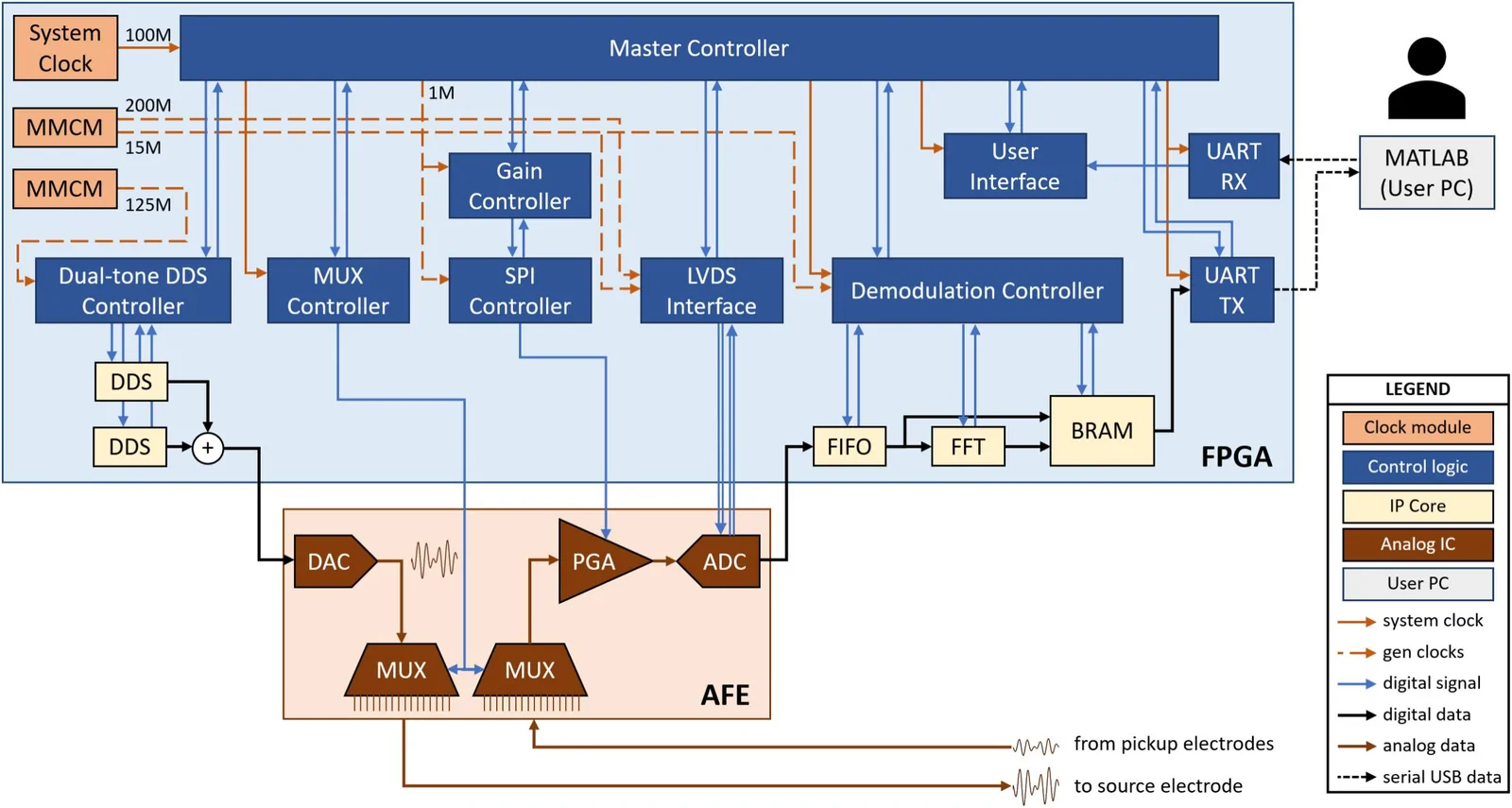
Block diagram of digital control modules and DIO to the AFE ICs. Clock signals are shown in orange: this includes a 100 MHz system clock (solid arrows) and several generated clocks for driving ADC and DAC DIO (dashed arrows). IP cores (yellow blocks) are controlled by higher-level controllers, and the master controller integrates all logic. Digital control signals are shown in blue and include logic signals to/from control modules and DIO to the DAC, ADC, MUX, and PGA. Black and red arrows denote the data path through digital and analog modules, respectively. (For interpretation of the references to colour in this figure legend, the reader is referred to the web version of this article.)

The FPGA firmware was developed in Very High Speed Integrated Circuit (VHSIC) Hardware Description Language (VHDL) code using Vivado 2022.2 (Xilinx, San Jose, CA) for synchronous control of the DAQ modules. An overview of the digital controllers implemented on the FPGA, as well as the cross-domain data path, is outlined in Fig. 9. DIO blocks for controlling the AFE ICs (PGAs, DAC, MUXs, and ADCs) have been implemented on the FPGA and interface the AFE through the 96-pin connectors on the FPGA carrier. A SPI bus is used for PGA control, parallel digital lines interface the DAC, static DIO lines control MUX switching and configure the ADCs, and a high-speed low-voltage differential signaling (LVDS) interface communicates with the ADCs. Mixed-mode clock managers (MMCMs) generate high-speed clocks for driving ADC and DAC DIO.

Xilinx Intellectual Property (IP) cores were leveraged when applicable. This design uses the DDS, Fast Fourier Transform (FFT), first-in-first-out buffer (FIFO), block memory (BRAM), and MMCM cores, and custom VHDL wrappers were developed to simplify high-level usage of these modules. Testbenches were written for each digital block, and all code was simulated in Vivado to validate the design logic prior to implementation on the FPGA. Finite state machines (FSMs) were developed to control the logic in each module. Sub-blocks provide handshake and control signals to higher-level blocks, and the top-level controller integrates all components with an FSM driven by user input decoded in a UART receiver block. The data path travels from the DDS source IP cores through the AFE, then back to the digital domain for demodulation (FIFO → FFT → BRAM IP cores).

Several clocks are generated to run the various components on the hardware, creating a complicated clock-domain-crossing (CDC) problem. A 100 MHz system clock runs the top-level and demodulation logic, a 125 MHz clock supplies DDS data to the DAC, a 200 MHz clock is used for LVDS communication with the ADC, and a 15 MHz clock is used to sample converted ADC data for the FFT. Signals running on all 4 clock speeds are used in the top-level controller, so CDC techniques such as flip-flop chaining (slow to fast clock), signal lengthening (fast to slow clock), and dual-clock FIFO usage have been employed to avoid metastability. In general, large data transfers across clock domains are achieved using FIFOs, and individual signals are stabilized with flip-flop chains or signal lengthening. As shown in Fig. 9, two MMCMs generate the clocks – one generates the 125 MHz DAC clock, and the other generates the 15 MHz ADC conversion and 200 MHz LVDS clocks to ensure phase synchronization of all clocked ADC DIO. The LVDS communication to the ADCs runs on a 200 MHz clock to achieve 400 MBPS data transfer, and the PGA SPI controller runs on a 1 MHz clock generated internally using a clock divider module. These high-speed signals require careful placement and routing during implementation to meet timing requirements; thus custom timing constraints were written to ensure the implemented design does not violate timing.

#### 3.3.1 Digital IO

DIO blocks for controlling the AFE ICs are implemented on the FPGA and interface the AFE through the two 96-pin connectors on the FPGA carrier. All DIO signals are 3.3 V except for the ADC signals – the LVDS signals require an IO bank with a 2.5 V reference voltage instead of 3.3 V; consequently, all other DIO on this IO bank (used to configure the ADC LVDS interfaces) have a high-level voltage of 2.5 V. A 1 MHz SPI interface is used to set PGA gain, and static DIO lines control MUX switching. Software commands from the MATLAB interface control MUX switching to provide more flexibility to the user than would be possible if implemented on hardware – selections available on the MATLAB GUI allow the user to specify settling time delays, Vs+/Vs- channel pairs, and voltage pickup channels, among several other acquisition parameters.

The forwarded clocks and high-speed signals require the use of specialized FPGA primitives implemented in the digital design, whereas other DIO signals use standard input and output buffers (IBUF and OBUF, respectively). On the input side, high-speed LVDS signals from the ADCs require differential signaling input buffers (IBUFDS), and the system clock is implemented using a dedicated global clock buffer BUFG. At the FPGA output, dual data rate output registers (ODDR) are instantiated for the clock signals forwarded to the DAC, ADCs, and PGAs, and differential signaling output buffers (OBUFDS) forward the LVDS clocks to the ADC interfaces. Since these dedicated buffers are fixed on the FPGA, the AFE design required careful consideration of DIO port locations to ensure the appropriate primitives for these signals were available on those pins. This is a critical design constraint that will need to be considered in the future when adding new probes or applications to the DAQ.

The complete digital design utilizes 45% of the Artix-7 FPGA IO buffers: DIO to the AFE is routed through 94 IOB (standard IO buffers) and 6 IBUFDS (differential buffers).

#### 3.3.2 Multi-tone source

The DDS IP Core (Xilinx) has been implemented for generating the voltage source sine wave. To generate a multi-tone source, a separate DDS core is instantiated for each frequency requested, and a digital controller synchronizes the blocks for proper phase alignment. The user sets frequency and phase offset parameters in MATLAB, which sends commands to the FPGA top-level logic. A lookup table (LUT) is used to set DDS phase bits to the requested frequency.

**Fig. 10:**
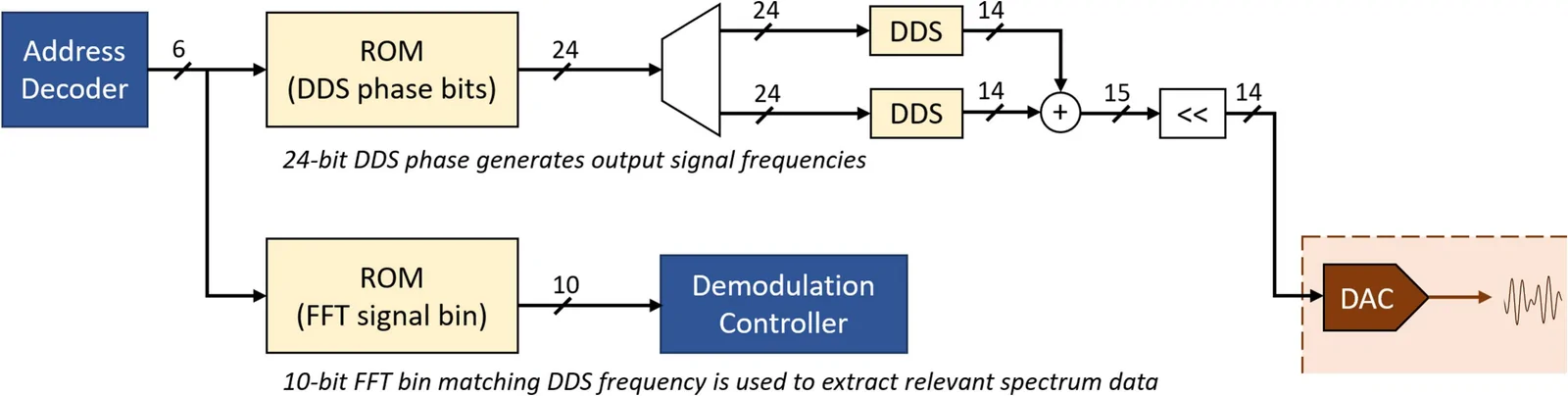
Simplified data path describes the dual-tone DDS implementation. In the top-level logic, a decoder selects one of 64 available frequencies stored in memory – the DDS phase bits that generate the output frequency are stored in one ROM, and the corresponding FFT bin for that frequency is in a second ROM. The decoder sets the memory address location, and the data is read to sub-modules (the DDS controller and demodulation controller) for additional processing.

A simplified data path describing the frequency selection logic is shown in Fig. 10. The address decoder sets the address location of two read-only memory (ROM) blocks, which are preloaded with matching DDS phase bits and FFT bins for 64 predetermined frequencies. Logic within the DDS controller reads the phase bits and selects the appropriate DDS core to set, allowing for either a single-tone or a dual-tone output. The FFT bin selection is sent to the demodulation controller, which uses this signal to extract the relevant FFT spectrum data as part of the demodulation scheme. The current design allows for a single-tone or dual-tone source to be generated by the DAC, but the number of tones can be increased with additional LUTs or DDS cores. However, it is important to note that adding tones ultimately reduces SNR, since the total signal amplitude is split between the generated sine waves (i.e., a single-tone source is a sine wave with amplitude A, whereas a dual-tone source will have a combination of two sine waves, each with amplitude A/2, resulting in an SNR drop of ∼3 dB).

The number of acquisition frequencies supported can be adjusted in the digital design by changing the size of the two ROM blocks and updating the corresponding coefficient files. The relationship of DDS frequency to FFT bin is not trivial due to the different clock speeds (the DAC runs on 100 or 125 MHz, whereas the ADC can only sample up to 15 MSPS) and data width limitations. The frequency resolution of the DDS core is determined by the clock speed ($f_{\mathrm{clk}}$, Hz) and number of phase bits (B). The output frequency ($f_{\mathrm{out}}$, Hz) is adjusted by changing the phase increment (Δθ), as described in (2). This design uses a 24-bit DDS core, resulting in a frequency resolution of 5.96 Hz when driven by a 100 MHz clock (or 7.45 Hz using the maximum clock speed of 125 MHz). The output frequency is determined by the 24-bit phase increment, which is set by the values stored in ROM:

(2) $$f_{\mathrm{out}}=\Delta \theta \frac{f_{\mathrm{clk}}}{2^{B}}$$

To determine the associated FFT bin (k) for the DDS output frequency, the FFT size (NFFT) and sampling rate ($f_{\mathrm{samp}}$, Hz) must be taken into consideration. Eq. (3) computes the DDS phase increment associated with the signal frequency in bin k of the FFT spectrum. The phase bits (Δθ(k)) and FFT bins (k) are stored in the top-level ROM blocks, as shown in Fig. 10.

(3) $$\Delta \theta \left(k\right)=k\frac{f_{\mathrm{samp}}}{\mathrm{NFFT}}*\frac{2^{B}}{f_{\mathrm{clk}}}$$

#### 3.3.3 Multi-tone demodulation

A 1024-point FFT has been implemented for multi-tone demodulation using the FFT IP Core (Xilinx). When demodulating multi-tone signals, the FFT provides more flexibility than a matched filter because it captures real and imaginary data for 512 (or NFFT/2) in-bin frequencies as opposed to one, and the full spectrum data can be used to easily calculate SNR and total harmonic distortion (THD). Furthermore, the number of possible in-bin acquisition frequencies can be increased by adjusting the sampling rate, as the frequency corresponding to each bin is dependent on the sampling rate.

A FIFO samples the ADC data at 15 MHz and transfers the data to the FFT and BRAM controllers at the 100 MHz system clock rate. The FFT core computes the forward transform, and then the real and imaginary spectrum data are used for pipelined, streaming computations during normal acquisition. All three ADCs (see Fig. 3) are sampled and demodulated in parallel, ensuring the relative phases are aligned for accurate complex demodulation (required for accurate EIT imaging), and synchronization of the blocks is driven by top-level logic. To maximize SNR, data averaging is employed (set by the user), and spectral leakage is minimized by matching the DDS frequency to an in-bin frequency for a given sampling rate via LUTs in the top-level controller. Demodulated data is stored in BRAM and transferred via UART to MATLAB for image reconstruction.

**Fig. 11:**
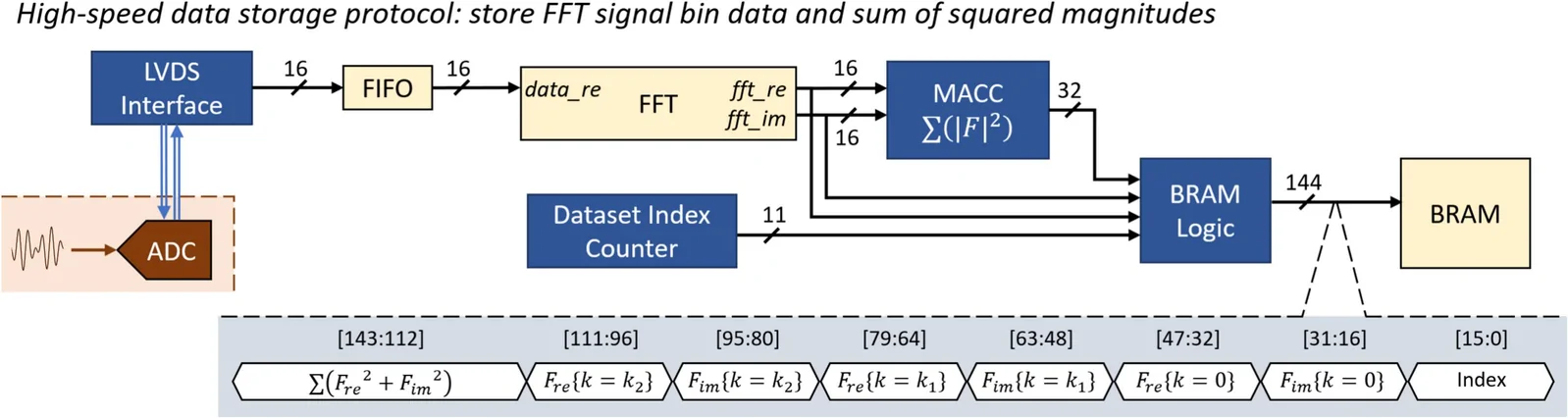
Diagram of the data path and data storage protocol implemented in the demodulation module. For high-speed acquisition, a summary of the important spectrum data in stored, which includes the sum of square magnitudes, signal bin data, DC bin data, and the dataset index.

The data storage protocol for high-speed acquisition is shown in Fig. 11 and involves storing a summary of the demodulated data, including FFT data for DC (bin 0) and DDS signal frequencies (bins k1 and k2 for dual-tone acquisition, or k1 and NFFT-k1 for single-tone), as well as the sum of squared magnitudes ∑(Re^2^ + Im^2^)) for computing SNR. Computations and logic are performed on the FFT spectrum data, and only one line of data is stored per dataset after the entire FFT spectrum has been read. The summarized data is stored in BRAM at the address equaling the dataset index, which is also saved to allow data transfer and parsing to be checked in software after acquisition is complete. The memory block has a depth of 2048, which is advantageous when a large number of averages or sequential datasets are desired to increase SNR or frame rate. When data averaging is implemented, each dataset is stored in a separate line in BRAM, and averaging is performed after all datasets have been acquired; this allows for easy validation of the user interface as additional functionality is developed.

#### 3.3.4 Acquisition timing

To achieve the targeted “real-time” imaging frame rate (30 fps), several system parameters need to be considered, as timing delays are required throughout the acquisition scheme. Data acquisition and demodulation logic on the FPGA require pipelining; data transfer between the FPGA and MATLAB is serially performed, and analog signals need time to stabilize when changes are made to the DDS frequency, MUX channels, or PGA gains. Thus, the achievable frame rate is inversely related to the number of frequencies acquired, measurements acquired, channels used, and averages computed, resulting in a tradeoff between attainable specifications (e.g., desired bandwidth, SNR, and frame rate). Table 2 shows the acquisition timing parameters used to determine frame rate. Acquisition time ($t_{\mathrm{acq}}$, s) for a single frame of data (ADC sampling and FFT demodulation) is described by (4), and the transmit time ($t_{\mathrm{tx}}$, s) to send a full dataset back to the user via UART is described by (5). For a measurement scheme with a specific number of source patterns ($N_{\mathrm{iis}}$), pickup patterns ($N_{\mathrm{vvs}}$), acquisition frequencies ($N_{f}$), and datasets per pattern/frequency combination ($N_{s}$), the total acquisition time ($t_{\mathrm{total}}$, s) can be calculated using (6). The N variables are dimensionless counts, and all time variables in (4)-(6) are expressed in seconds.

(4) $$t_{\mathrm{acq}}=t_{\mathrm{sample}}+t_{fft\_input}+t_{fft\_compute}+t_{fft\_output}+t_{save\_fft}$$

(5) $$t_{\mathrm{tx}}=\left(N_{\mathrm{adcs}}\times \frac{N_{data\_bits}}{8}\times N_{uart\_bits}\times t_{\mathrm{baudrate}}\right)+t_{freq\_delay}$$

(6) $$\begin{array}{c} t_{\mathrm{total}}=\left(t_{set\_iivv}\times N_{\mathrm{iis}}\right)+\left(t_{set\_vv}\times N_{\mathrm{iis}}\times \left(N_{\mathrm{vvs}}-1\right)\right)+\\ \left(t_{freq\_delay}\times N_{\mathrm{iis}}\times N_{\mathrm{vvs}}\times \left(N_{f}-1\right)\right)+\left(N_{s}\times \left(t_{\mathrm{acq}}+t_{\mathrm{tx}}\right)\right) \end{array}$$

For a fixed set of source and pickup channels (standard tetrapolar EIT configuration), the time required to sweep all 64 preselected frequencies is dominated by the low-frequency ADC sampling time. The lower 32 frequencies (100 Hz to 24.3 kHz) are sampled on the slow ADC clock, while the higher 32 frequencies (14 kHz to 3 MHz) are sampled on the fast ADC clock. The total acquisition time (ADC acquisition and FFT processing) for the 32 lower frequencies is 294.3 ms, while the higher 32 frequencies require 3.0 ms. The data transfer back to the user (t_data_tx_) occurs after all data is acquired and saved in FPGA memory. Thus, the total acquisition time for a full 64-frequency sweep is approximately 299 ms for a tetrapolar configuration, including the initial 430 µs MUX configuration and 773 µs final data transfer. More generally, for $N_{\mathrm{iivvs}}$ arbitrary source/pickup patterns, $N_{f}$ frequencies, and $N_{s}$ datasets (including datasets used for averaging), frame acquisition time ($t_{\mathrm{frame}}$, s) can be calculated by (7), where frame rate ($f_{\mathrm{frame}}$, Hz) is the reciprocal (8). When using a combination of low and high frequencies ($N_{\mathrm{lf}}$ and $N_{\mathrm{hf}}$, respectively), the frame acquisition time (4) includes the slow and fast ADC $t_{\mathrm{sample}}$ and can be calculated by (9).

(7) $$t_{\mathrm{frame}}=N_{\mathrm{iivvs}}\left(t_{mux\_delay}+N_{f}\times N_{s}\times t_{\mathrm{acq}}+\left(N_{f}-1\right)\times t_{freq\_delay}\right)+t_{data\_tx}$$

(8) $$f_{\mathrm{frame}}=\frac{1}{t_{\mathrm{frame}}}$$

(9) $$t_{\mathrm{frame}}=N_{\mathrm{iivvs}}\left(t_{mux\_delay}+N_{s}\left(N_{\mathrm{lf}}\times t_{\mathrm{acq},slow}+N_{\mathrm{hf}}\times t_{\mathrm{acq},fast}\right)+\left(N_{\mathrm{lf}}+N_{\mathrm{hf}}-1\right)\times t_{freq\_delay}\right)+t_{data\_tx}$$

The frame rate specification does put a limit on frequency switching, averaging (i.e., lower SNR), and MUX switching (i.e., fewer channels), but the use of dual-tone acquisition can allow two frequencies to be acquired simultaneously without switching, a major benefit of this system’s design. Depending on the imaging protocol, several acquisition strategies satisfy the 30 fps design target:

• If a frequency sweep is desired (e.g., spectroscopy measurements), it is recommended to exclude the lower frequencies to meet “real-time” imaging requirements—with this configuration, one can acquire 32 frequencies (14 kHz to 3 MHz) for 8 arbitrary source/pickup channel combinations at 30 fps.
• If only one frequency (or one dual-tone acquisition) is required and the slow ADC sampling time is used (frequency range 100 Hz to 24.3 kHz), the 30 fps target can be met with 3 source/pickup channel combinations.
• If only one frequency (or one dual-tone acquisition) is required and the fast ADC sampling time is used (frequency range 14 kHz to 3 MHz), the 30 fps target can be met with 62 source/pickup channel combinations.

**Table 2:** Acquisition timing parameters.

Name	Description	Clock cycles	Clock period (ns)	Time (µs)
t_uart_tx_	UART packet transfer	11	3906	43
t_sample_ (min)	Sample ADC frame (fast)	1024	70	71.7
t_sample_ (max)	Sample ADC frame (slow)	1024	8960	9175
t_fft_input_	Send ADC frame to FFT input	1024	10	10.24
t_fft_compute_	Perform FFT	112	10	1.12
t_fft_output_	Streaming FFT spectrum output	1024	10	10.24
t_save_fft_	Save FFT spectrum data	5	10	0.05
t_mux_delay_	MUX switch delay	5000	10	50
t_freq_delay_	Frequency switch delay	1024	10	10.24
t_set_iivv_	Set MUX channels (Vs+, Vs-, V1, V2)	10 x t_uart_tx_	3906	430
t_set_vv_	Set MUX pickup channels (V1, V2)	6 x t_uart_tx_	3906	258
t_data_tx_ (typ)	Send data to user (high-speed mode)	18 x t_uart_tx_	3906	773
t_data_tx_ (max)	Send data to user (debug mode)	4098 x t_uart_tx_	3906	176,075

These metrics assume no dataset averaging is included in the acquisition sequence (averaging is an optional feature used to increase SNR in noisy environments). Because the source/pickup switching delays and UART transfer occur only once per measurement, dataset averaging scales only the acquisition portion of the timing rather than the entire acquisition sequence. Consequently, averaging has a smaller impact on frame rate than increasing the number of channel configurations or frequency steps. For example, using four-dataset averaging on a single-frequency acquisition reduces the maximum number of source/pickup channel combinations from 62 to 40 while still maintaining the 30 fps target.

Given the current FPGA implementation (no dataset averaging), the 30 fps target can be met for several standard EIT setups, including single- or dual-frequency 16-channel single skip pattern measurements (16 patterns) for lung imaging, as well as tetrapolar impedance spectroscopy. The acquisition speed for 64 unique source/pickup patterns (for single- or dual-tone acquisition > 14 kHz) is 29.2 fps, slightly under the target rate. These results demonstrate the trade-off between spectral resolution, channel count, averaging, and frame rate; applications requiring broadband impedance spectroscopy are limited primarily by the low-frequency acquisition time, whereas applications operating above 14 kHz can include additional electrode channels and dataset averaging while maintaining real-time imaging performance.

#### 3.3.5 Software user interface

A GUI was developed in MATLAB to provide a user-friendly approach for operating the DAQ and visualizing performance. The user sets acquisition parameters in the GUI, and the associated functions are executed to send UART commands to the FPGA for system configuration and measurement trigger. A UART receiver reads the demodulated data sent by the FPGA as it becomes available. Once acquisition and data transfer are complete, the data is parsed, extracted into complex impedances, and displayed in the GUI. Manual control of DDS frequencies, MUX channels, PGA gains, and ADC configuration is available with simple function calls, which is useful for calibration and testing.

The acquisition sequence consists of nested loops for acquiring multi-channel and multi-frequency data, and parameter switching is controlled by both software commands and hardware-defined logic. The loop order from outermost to innermost is:

1. Set source MUX channel pair
2. Set pickup MUX channel pair
3. Set frequency(s)
4. Acquire data
5. Send data back to the user.

**Fig. 12:**
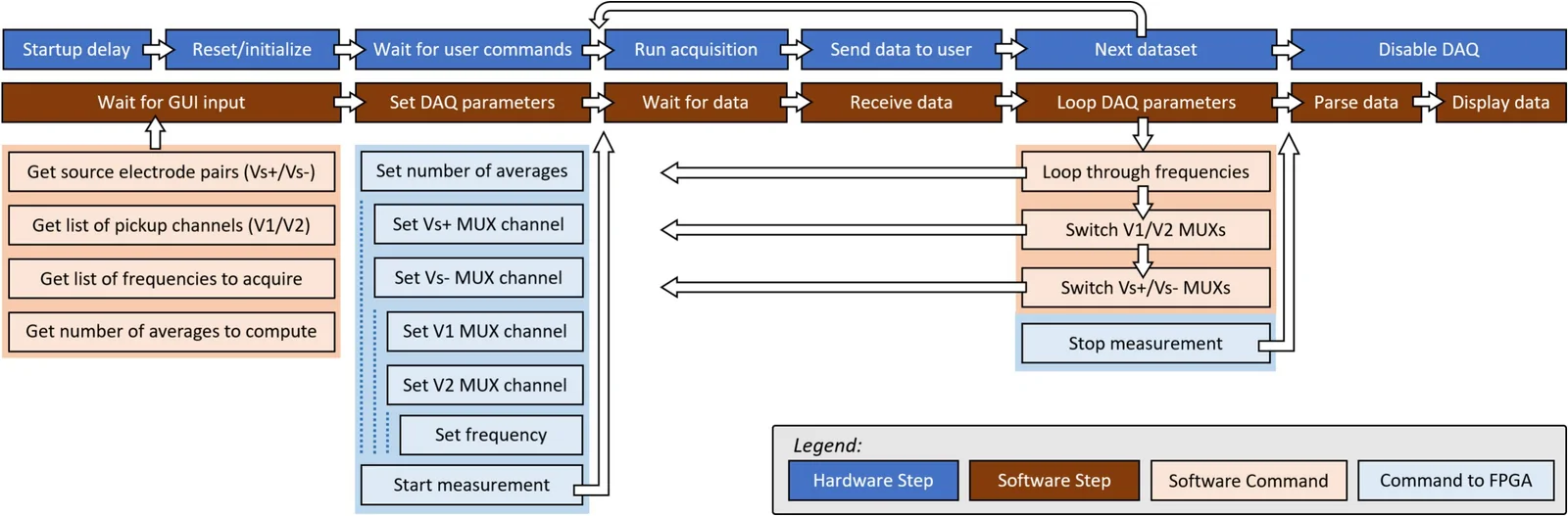
High-level block diagram of the acquisition logic. After the initial startup delay, hardware steps are driven by commands sent from the software UI. Internal software commands load acquisition parameters from the GUI and trigger frequency and MUX channel switching.

MUX channel switching logic (i.e., looping through user-defined source pairs and pickup channels) is controlled in software, whereas frequency switching is automated in hardware to minimize delays stemming from serial communication occurring during acquisition. A high-level description of the acquisition sequence is depicted in Fig. 12. After the initial startup delay, hardware parameter settings and measurement trigger are driven by the software UI. Internal software commands load acquisition parameters from the GUI and drive the switching logic (e.g., MUX channel switching), and commands sent to the FPGA control hardware settings for each measurement. After acquisition is complete, the top-level controller goes into a low-powered state in which nonessential modules are disabled (e.g., FFT controller, DDS output, LVDS interface). The sequence restarts at the “reset” step if subsequent measurements are triggered without powering the system off.

## 4 Design files summary

**Table:** 

Design file name	File type	Open source license	Location of the file
EIT-DAQ-BOM-Details.xlsx	Bill of materials	CERN-OHL-W-2.0	https://doi.org/10.5281/zenodo.21359298
EIT-AFE-Schematics.pdf	Schematic	CERN-OHL-W-2.0	https://doi.org/10.5281/zenodo.21359298
EIT-AFE-Gerber.zip	PCB Gerber	CERN-OHL-W-2.0	https://doi.org/10.5281/zenodo.21359298
64Ch-MUX-AFE-Schematics.pdf	Schematic	CERN-OHL-W-2.0	https://doi.org/10.5281/zenodo.21359298
64Ch-MUX-AFE-Gerber.zip	PCB Gerber	CERN-OHL-W-2.0	https://doi.org/10.5281/zenodo.21359298
eit_daq_top_design.bit	FPGA bitstream	CERN-OHL-W-2.0	https://doi.org/10.5281/zenodo.21359298
eit_daq_mem_config.bin	FPGA memory configuration	CERN-OHL-W-2.0	https://doi.org/10.5281/zenodo.21359298
EIT-DAQ-Vivado-Project.zip	Vivado/VHDL source files	MIT License	https://doi.org/10.5281/zenodo.21359298
EIT-DAQ-MATLAB-UI.zip	MATLAB source files	MIT License	https://doi.org/10.5281/zenodo.21359298

• **EIT-DAQ-BOM-Details.xlsx** – bill of materials summary with circuit designators for each PCB assembly
• **EIT-AFE-Schematics.pdf** – circuit schematics for the base EIT AFE PCB
• **EIT-AFE-Gerber.zip** – Gerber files for the base EIT AFE PCB
• **64Ch-MUX-AFE-Schematics.pdf** – circuit schematics for the 64-channel MUX AFE PCB
• **64Ch-MUX-AFE-Gerber.zip** – Gerber files for the 64-channel MUX AFE PCB
• **eit_daq_top_design.bit** – Vivado-generated bitstream file with Artix-7 FPGA hardware design. Use this file to program the board through Joint Test Action Group (JTAG).
• **eit_daq_mem_config.bin** – Vivado-generated binary file with the memory configuration for programming the board through Quad-SPI (QSPI). This file will load the QSPI flash memory with the eit_daq_top_design.bit bitstream.
• **EIT-DAQ-Vivado-Project.zip** – Vivado project and VHDL source files. These are not required for the build and operation of the system as provided, but they can be modified if additional functionality is desired. Use the TCL script to recreate the full project.
• **EIT-DAQ-MATLAB-UI.zip** – MATLAB software user interface source files

## 5 Bill of materials summary

The bill of materials has been provided in the Supplementary Material. The spreadsheet (*EIT-DAQ-BOM-Details.xlsx)* details all components used on the two custom-designed PCBs, as well as the Trenz Electronic FPGA development boards.

## 6 Build instructions

### 6.1 Hardware assembly

**Fig. 13:**
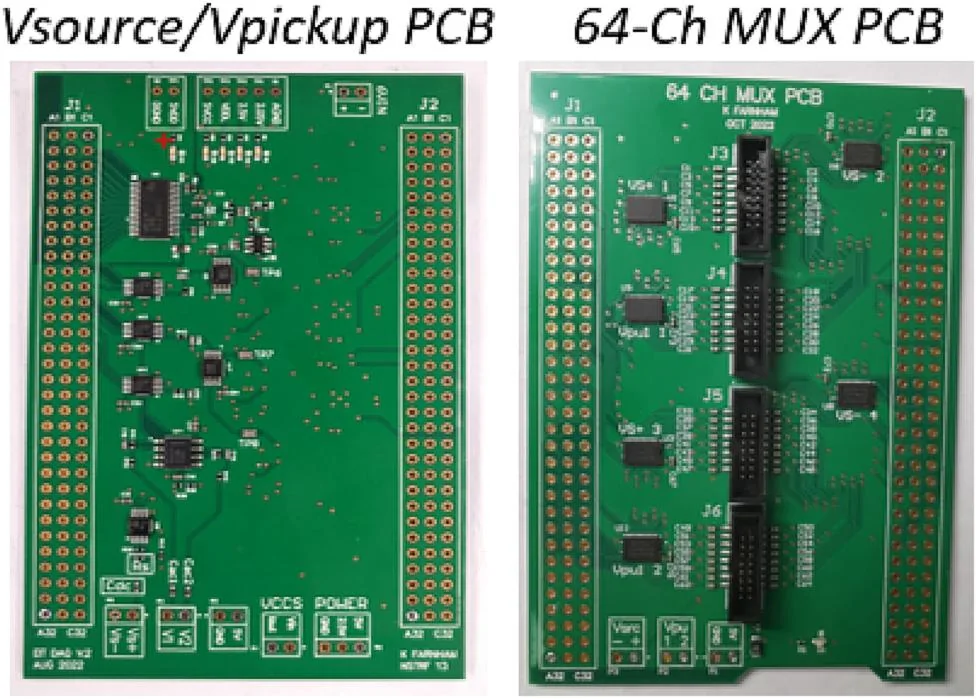
The two PCB assemblies that comprise the custom AFE. The integrated voltage source and pickup PCB (left) and 64-channel MUX PCB (right) stack on top of an Artix-7 (Xilinx) FPGA carrier board via 96-pin connectors.

Two custom PCBs make up the 64-channel AFE and can be reproduced from the Gerber files provided. The AFE is divided into two PCBs to conserve board space and provide an adaptable electrode interface: one PCB houses the voltage source and pickup circuits (*EIT-AFE-Gerber.zip*), while the other implements a 64-channel MUX interface to the electrodes (*64Ch-MUX-AFE-Gerber.zip*). PCBWay manufactured and assembled the dual-sided PCBs shown in Fig. 13. The discrete components and designators for each PCB assembly are provided in the Bill of Materials summary (*EIT-DAQ-BOM-Details.xlsx)*. Components can be purchased directly from Digikey or sourced through the PCB manufacturer.

**Fig. 14:**
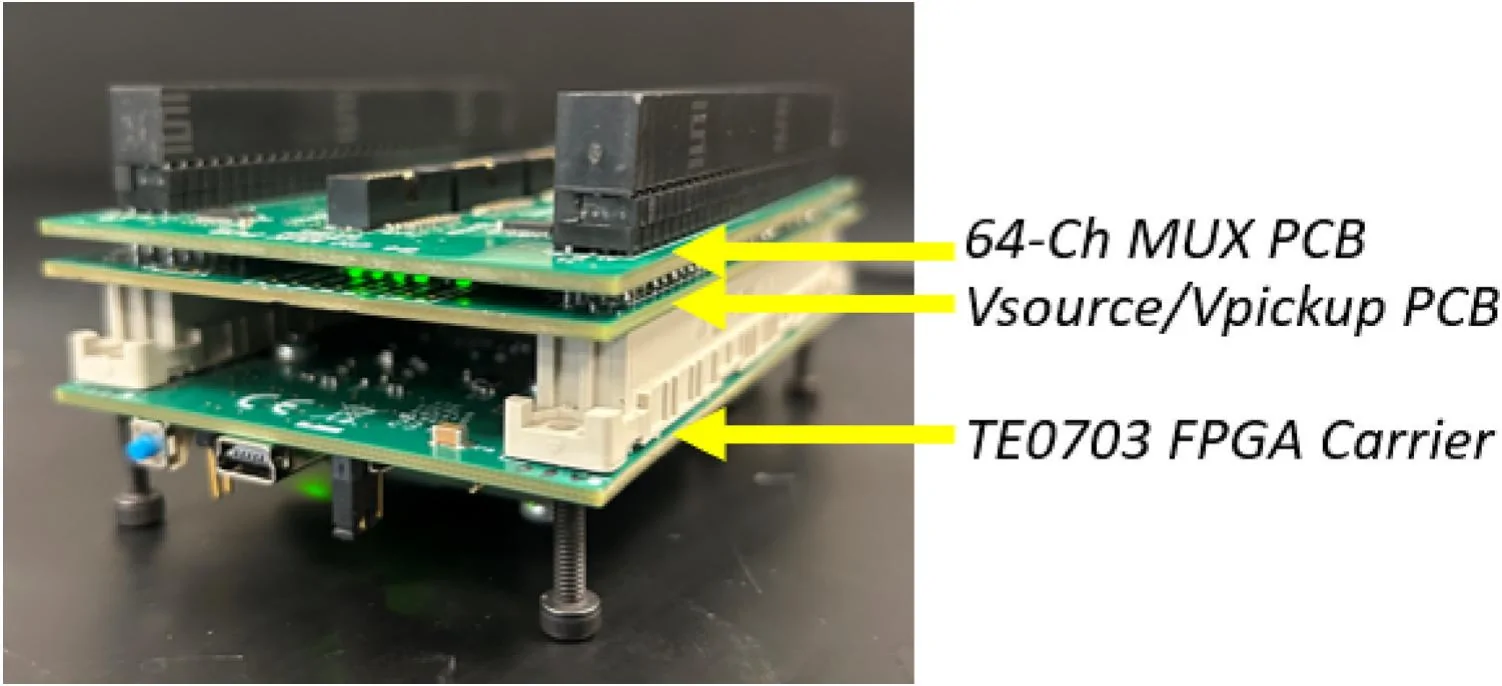
The assembled board stack includes the Trenz Electronic carrier (TE0703-06), the voltage source and pickup PCB, and the 64-channel MUX PCB. The full system size is 10 × 8 × 8 cm.

The FPGA carrier boards (TE0703 and TE0711) from Trenz Electronic provide a base for the DAQ board stack with built-in 96-pin connectors. These boards are also listed in the Bill of Materials, and can be purchased through Digikey or Trenz Electronic. Once the custom PCBs are soldered and assembled, the AFE stacks on top of the TE0703, resulting in a full system size of 10x8x8 cm. The full system stack should include (from bottom to top) the FPGA carrier (TE0703-06) with the FPGA module (TE0711-01), the voltage source/pickup PCB, and the 64-channel electrode interface PCB (Fig. 14).

**Fig. 15:**
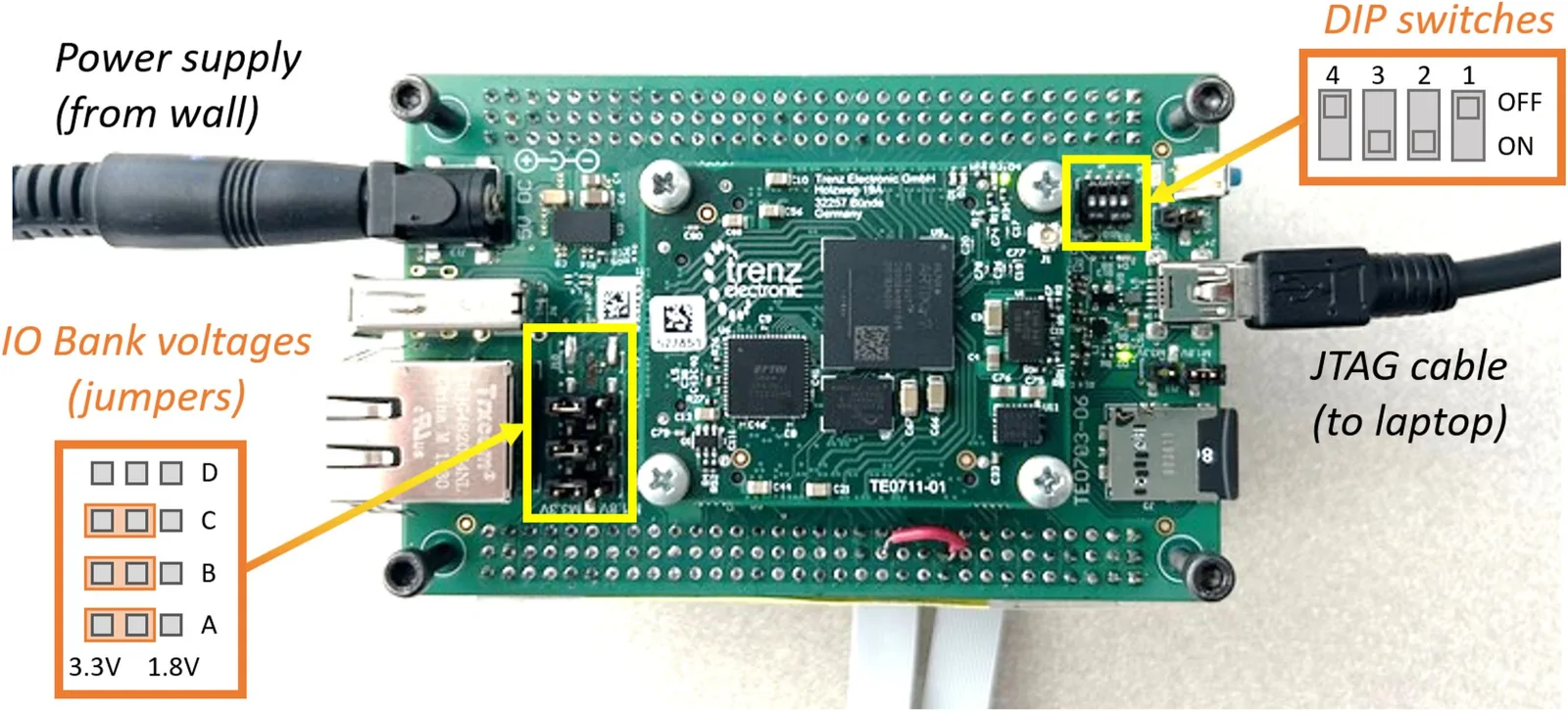
The bottom of the TE0703 carrier board contains peripherals used for configuration. Set the FPGA IO bank voltages using the jumpers, configure the JTAG and FPGA programming method with the DIP switches, and use the JTAG cable to load the bitstream file onto the FPGA from Vivado. Set all four DIP switches ON if programming through JTAG; otherwise, set switches 1 and 4 OFF as shown. The power supply and JTAG cable connect to the DAQ via the TE0703 as well.

To configure the TE0703 carrier for DAQ operation, set the IO Bank voltage and DIP switches appropriately:

1. Set jumpers J5, J8, and J9 to 3.3 V, and remove jumper J10. This will set three of the FPGA IO bank voltages (A, B, and C) to 3.3 V, as seen in Fig. 15. The fourth (D) will be set to 2.5 V externally using a jumper wire (Fig. 16).

**Fig. 16:**
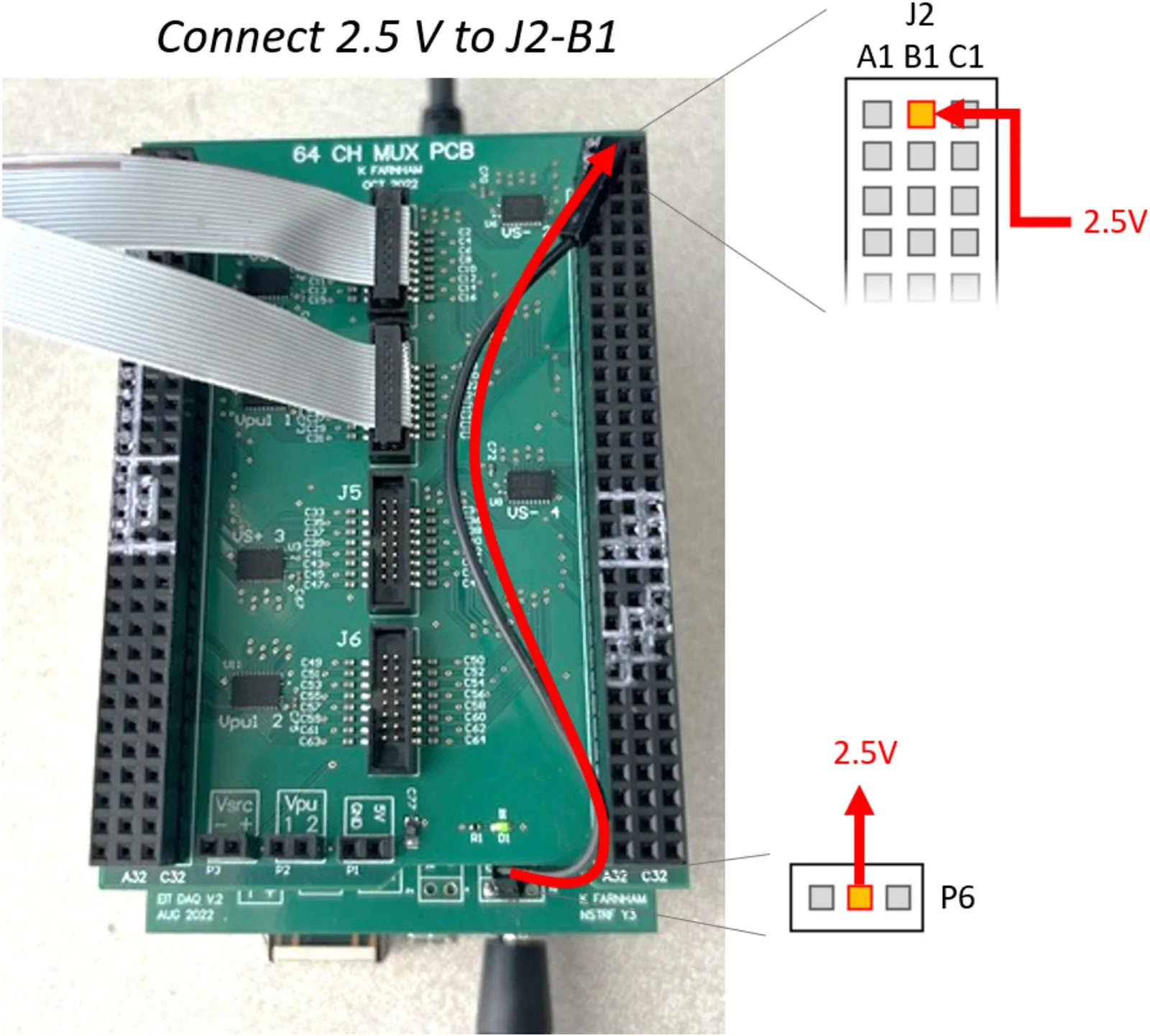
The FPGA IO bank voltage for the ADC DIO must be set to 2.5 V for proper operation. Use the 2.5 V port on the voltage source/pickup PCB as the source, and connect this to pin J2-B1 to set the IO bank voltage. This wire is highlighted in yellow in the figure. CAUTION: Ensure jumper J10 on TE0703 is disconnected prior to connecting any external voltage to pin J2-B1. (For interpretation of the references to colour in this figure legend, the reader is referred to the web version of this article.)
2. To set the IO Bank D voltage externally, use a jumper wire to connect 2.5 V up to J2-B1, as shown in Fig. 16. This will provide the 2.5 V reference for the ADC DIO pins. (Caution: ensure the jumper on J10 is disconnected before connecting J2-B1 to 2.5 V).
3. Set the DIP switches S2-2 and S2-3 to the ON position. Switches S2-1 and S2-4 set the programming method. If programming directly through JTAG, switch these ON. If programming through the memory configuration file, set both switches (1 and 4) OFF (Fig. 15)

### 6.2 Programming the FPGA

Once the TE0703 is configured, the FPGA needs to be programmed through an external laptop running Vivado 2024.1. Download the hardware design files from the Zenodo repository. Connect the TE0703 board to the external laptop via a JTAG cable, open Vivado, and connect to the board using the Vivado *Hardware Manager*. There are two ways to program the Artix-7 FPGA:

1. JTAG, for volatile programming (i.e., the configuration will be lost when the FPGA is powered off). This method is recommended if making alterations to the design that require testing and validation.
2. QSPI, for persistent programming (i.e., the configuration is saved and will reboot when the FPGA is power cycled). This method is recommended for repeated use of the final design.

To program through JTAG, load the bitstream file provided (*eit_daq_top_design.bit*) using the *Hardware Manager*. Make sure all the DIP switches on the TE0703 are set to ON.

To program the Artix-7 FPGA for repeated use after power cycling, the QSPI method must be used. The QSPI settings are located in the Vivado project in the *bitstream_config.xdc* constraints file, and the binary memory configuration file is provided as *eit_daq_mem_config.bin*.

1. Before programming, set the TE0703 DIP switches 1 and 4 to the OFF position (see Fig. 15).
2. Open the *Hardware Manager* and connect to the board.
  a. Right-click on the part xc7a100t, and select *Add Configuration Memory Device*.
  b. Set the memory part number to S25FL256S. This is the QSPI part number found on the TE0711-01 datasheet [29].
3. Program the configuration memory device using the provided binary file *eit_daq_mem_config.bin*.
4. Close the *Hardware Manager*.
5. Power cycle the board to boot from QSPI. NOTE: If the Hardware Manager is open when the board is power cycled, the FPGA will not boot from QSPI.

#### 6.2.1 Create the Vivado project

To recreate the full Vivado project for adding customizations, open the *TCL Console* in Vivado 2024.1. This step is only necessary if making alterations to the FPGA design.

1. Unzip the *EIT-DAQ-Vivado-Project.zip* folder provided in the Zenodo repository—this contains the TCL script and source files to recreate the project. Place the files in a location that has no spaces in the path.

2. In the TCL Console, use the **cd** command to change directories to the location of the *2025_DAQ.tcl* script, then run the script using the **source** command. For example:

cd C:/Users/d33230f/Downloads/EIT-DAQ-Vivado-Project/

source./2025_DAQ.tcl

3. The project will be created in the source directory and named 2025_DAQ by default.

#### 6.2.2 Regenerating the memory configuration files

If customizations are made to the Vivado design (or if a different version of Vivado is used), a new memory configuration file must be generated in Vivado.

1. Open the Vivado project, and regenerate the bitstream by clicking *Generate Bitstream* in the *Flow Navigator* panel.
2. Once the new bitstream has been generated, a new memory configuration file must be created for QSPI flash. In the top menu, navigate to *Tools > Generate Memory Configuration File*.
  a. Use the drop-down to set the *Format* to *BIN*.
  b. Set the memory part number to S25FL256S, the QSPI part on the TE0711-01 [29].
  c. Create a filename for the binary file.
  d. Change the interface to *SPIx4*.
  e. Click the *Load bitstream files* checkbox, and navigate to the generated bitstream file.
  f. Click *OK* to save these settings.
3. Open the *Hardware Manager* and connect to the board.
  a. Right-click on the part xc7a100t, and select *Add Configuration Memory Device*.
  b. Set the memory part number to S25FL256S, same as the memory configuration file.
4. Program the configuration memory device, and select the binary file generated during configuration.
5. Close the *Hardware Manager* prior to power cycling. NOTE: If the Hardware Manager is open when the board is power cycled, the FPGA will not boot from QSPI. Make sure the TE0703 DIP switches 1 and 4 are set to the OFF position.

### 6.3 Software

Download the software used to run the DAQ from the Zenodo repository (*EIT-DAQ-MATLAB-UI.zip*). A MATLAB GUI is provided as a MATLAB App (*main_gui.mlapp*) and can be customized as needed. All software functions are included in the *mfiles* directory within the repository. This software was developed for MATLAB 2023b, but should be compatible with other versions.

## 7 Operation instructions

**Fig. 17:**
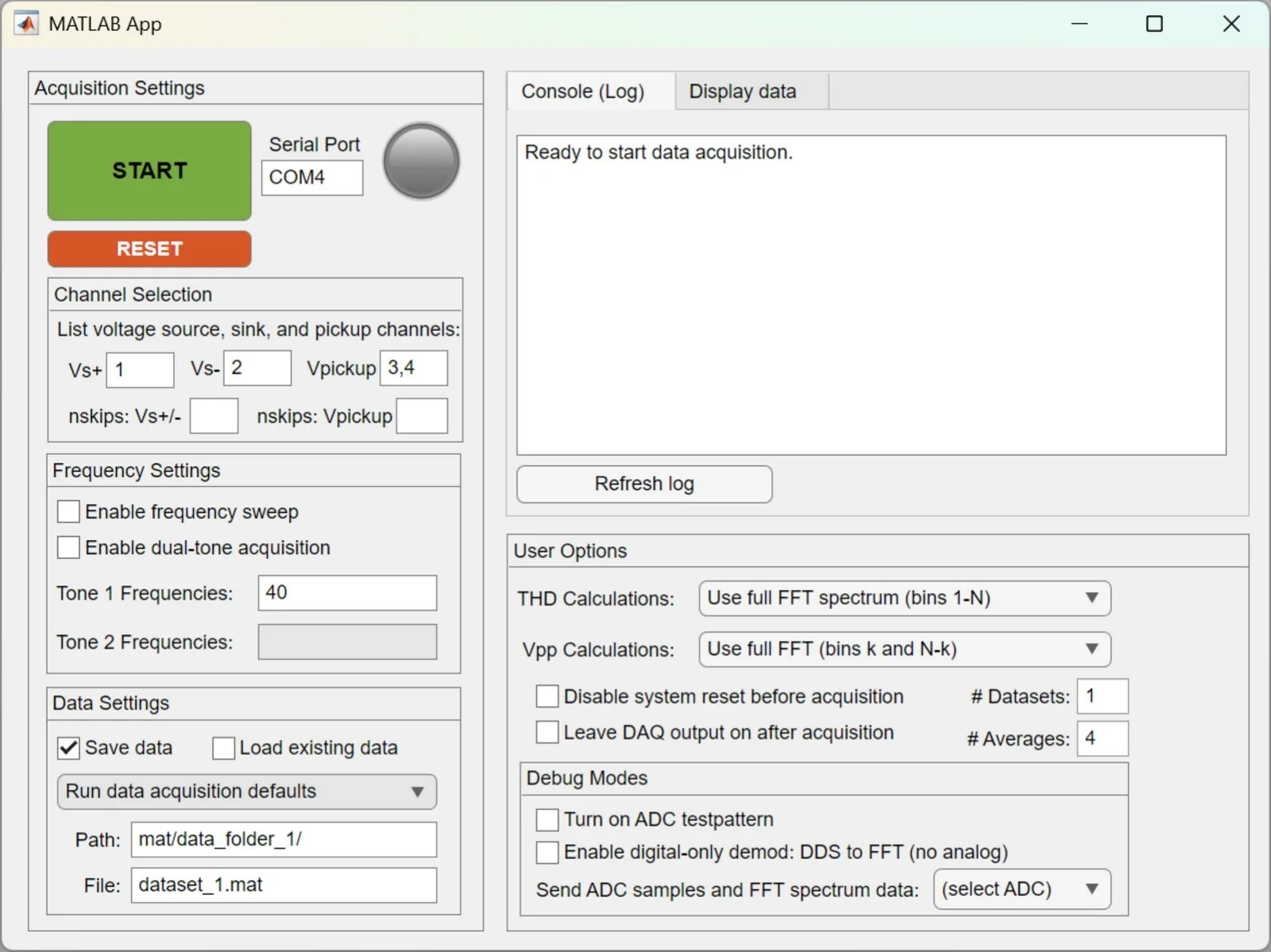
MATLAB App developed for the FPGA-based DAQ GUI.

The DAQ operation requires an external 5 V DC power supply, a JTAG cable, and an external laptop with a MATLAB installation for running the GUI. The TE0703 carrier provides the power jack and JTAG connector header for FPGA programming and data transfer (Fig. 15). To turn on the system, plug the 5 V power supply into the power jack on the TE0703 carrier. Connect the DAQ to the operator laptop using a JTAG to USB cable. Open MATLAB, and run the MATLAB GUI (*main_gui.mlapp*) provided in the software folder (*EIT-DAQ-MATLAB-UI.zip*) of the Zenodo repository. The startup GUI is shown in Fig. 17.

To run the DAQ, enter the acquisition parameters in the GUI (Fig. 17) and click the “Start” button to begin the measurement. Software commands drive the acquisition sequence and parameter switching (refer to Fig. 12 for the full acquisition sequence). After the measurement is complete, software functions (located in the *mfiles* folder) parse the serial data stream into usable data based on the data transfer protocol selected and plot the data in the GUI display panel per user selections.

**Fig. 18:**
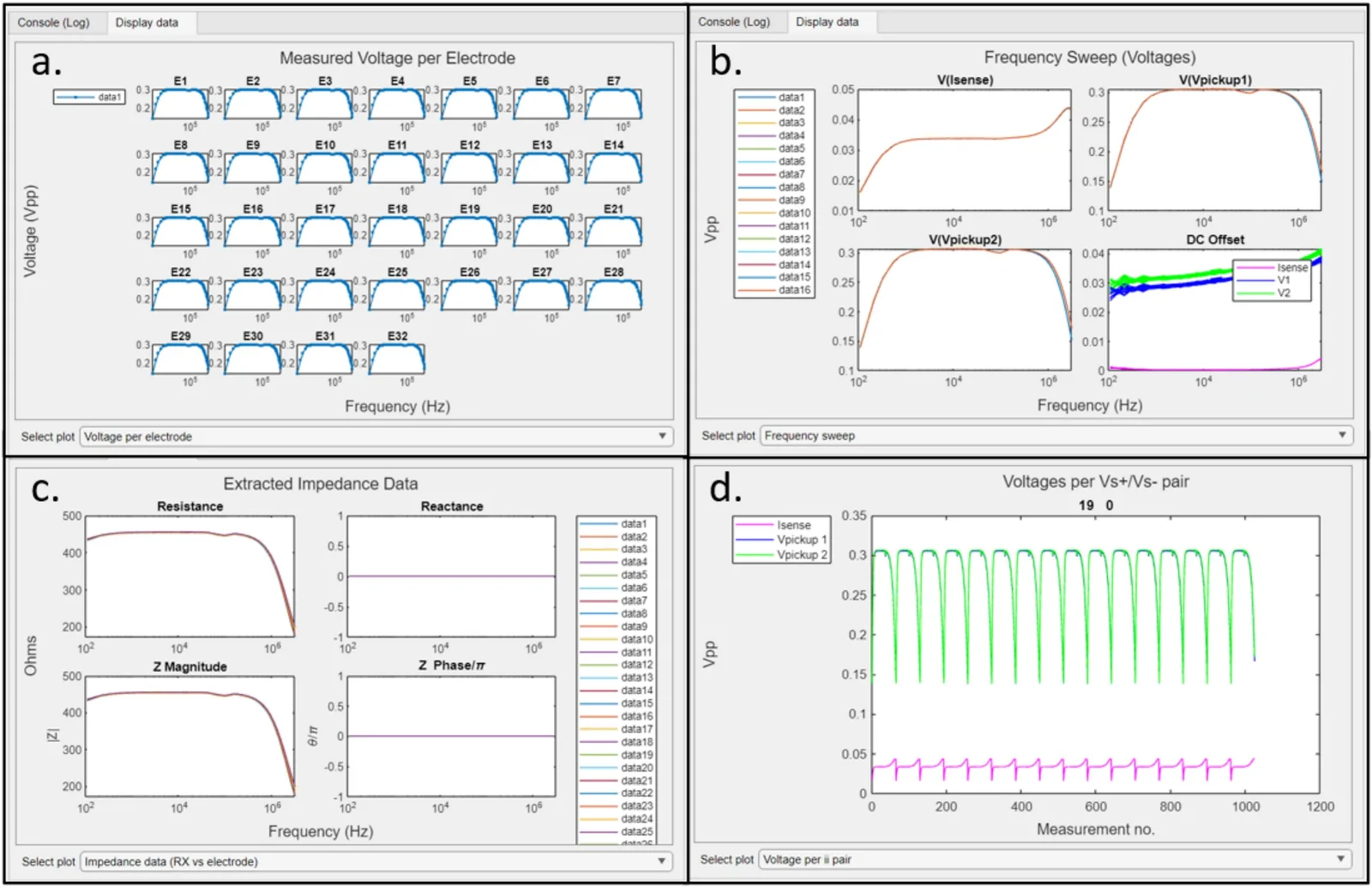
The drop-down menu on the GUI display panel offers several ways to view the data. This dataset was used to assess channel-to-channel variation: frequency sweep data (64 frequencies 100 Hz–3 MHz) was acquired for a single Vs+/- pair driving 32 channels attached to a discrete load. (a) Individual plots for each of the 32 measurement channels show voltage curves over frequency. (b) All 16 voltage measurements from the Visense, Vpickup1, and Vpickup2 ADCs are plotted vs. frequency. (c) Complex impedance data computed using the voltage and current sense data is plotted vs. frequency for each of the 32 measurements. (d) Voltage measurements from all 3 ADCs are displayed over time.

The GUI provides a variety of plots for data visualization. Fig. 18 shows four example plot types available from the drop-down menu on the GUI display panel, accessed by clicking the “Display data” tab on the main GUI. The data in Fig. 18 shows frequency sweep data (64 frequencies, 100 Hz – 3 MHz) acquired from 32 channels: the 32 voltage pickup channels are attached to a discrete resistive load, and a single Vs+/- pair drives the source. Because all 32 pickup channels are attached to the same load, we should expect the measured voltages to all be the same and the measured current to stay constant while pickup channels are switched (i.e., this circuit configuration can be used to assess channel-to-channel variation). Indeed, all 32 pickup datasets have nearly equivalent frequency responses and measured impedance magnitudes, and the frequency response of the sensed current stays constant throughout as well. Plots like these are extremely useful for verifying that the system is operating as expected, such as detecting a “bad” channel that may have poor electrode contact.

## 8 Validation and characterization

### 8.1 Hardware evaluation

The EIT DAQ was evaluated over frequencies 100 Hz to 3 MHz using discrete resistive loads ranging from 10 Ω to 10 kΩ. Table 3 summarizes the final performance of the hardware with respect to the targets previously listed in Table 1. The DAQ can meet all target specifications, but it is critical to note the trade-offs involved. For instance, frame rate is inversely dependent on the number of frequencies acquired, measurements acquired, channels used, and averages computed; thus, increasing any of these parameters may consequently slow the frame rate to less than the targeted 30 fps (see Section 2.3.4*. Acquisition Timing* for more details). The acquisition settings used for assessing DAQ performance yield much slower frame rates (<1 fps), with 64 frequencies (100 Hz to 3 MHz), 1024 demodulation samples, and 4 averages computed per measurement. For most EIT applications, frame rate can be reduced without any repercussions, as the imaging subject is mostly static (e.g., cancer imaging, internal bleeding detection, muscle imaging). A high frame rate is necessary only when imaging high-speed dynamic systems, such as pulmonary or cardiac function monitoring—in these cases, the 30 fps frame rate can be achieved by reducing the number of channels to 16 and limiting the number of frequencies acquired to four or less.

**Table 3:** DAQ hardware performance summary.

Specification	Target	Final system performance
Frame rate	> 30 fps	> 30 fps achievable with a variety of measurement conditions (see Section 2.3.4*. Acquisition Timing* for full list of possible imaging setups for 30 fps)
Impedance precision	1 Ω	< 1 Ω for loads ≤ 3 kΩ, < 1.3 Ω for loads ≤ 10 kΩ
Impedance range	10 Ω–3 kΩ, 1 pF–30 nF	Characterized for resistive loads 10 Ω–10 kΩ
Bandwidth	100 Hz–1 MHz	Characterized for frequencies 100 Hz–3 MHz
No. channels	64	64
SNR	80 dB	>80 dB for loads > 50 Ω and frequencies 1 kHz–1 MHz with 4-dataset averaging (or 60–70 dB without dataset averaging)
Max current	<10 mA	<10 mA, adjustable
Power supply	Wall power	5 V AC/DC wall supply
Size	<12 × 12 × 12 cm	10 × 8 × 8 cm
Mass	<1 kg	0.3 kg
User interface	MATLAB GUI	MATLAB GUI

SNR (dB) was computed over 100 consecutive measurements using (10), where µ is the mean, and σ is the standard deviation computed using MATLAB’s std function.

(10) SNR=20log10μσ

The 80 dB SNR target specification can be attained through data averaging or by increasing the amplitude of the voltage source, as smaller amplitude signals require more averaging to achieve the same SNR as larger signals. Additionally, the mismatch between DDS frequency and expected FFT frequency is greatest at low frequencies due to quantization error from the bit-limited DDS phase control, causing spectral leakage and consequent degradation in SNR.

Increasing the number of bits used in the DDS core would alleviate this issue at the expense of FPGA fabric usage and more complex routing to meet timing constraints – this is a trivial adjustment for future revisions of the system, as the hardware design can be easily ported to a larger FPGA.

**Fig. 19:**
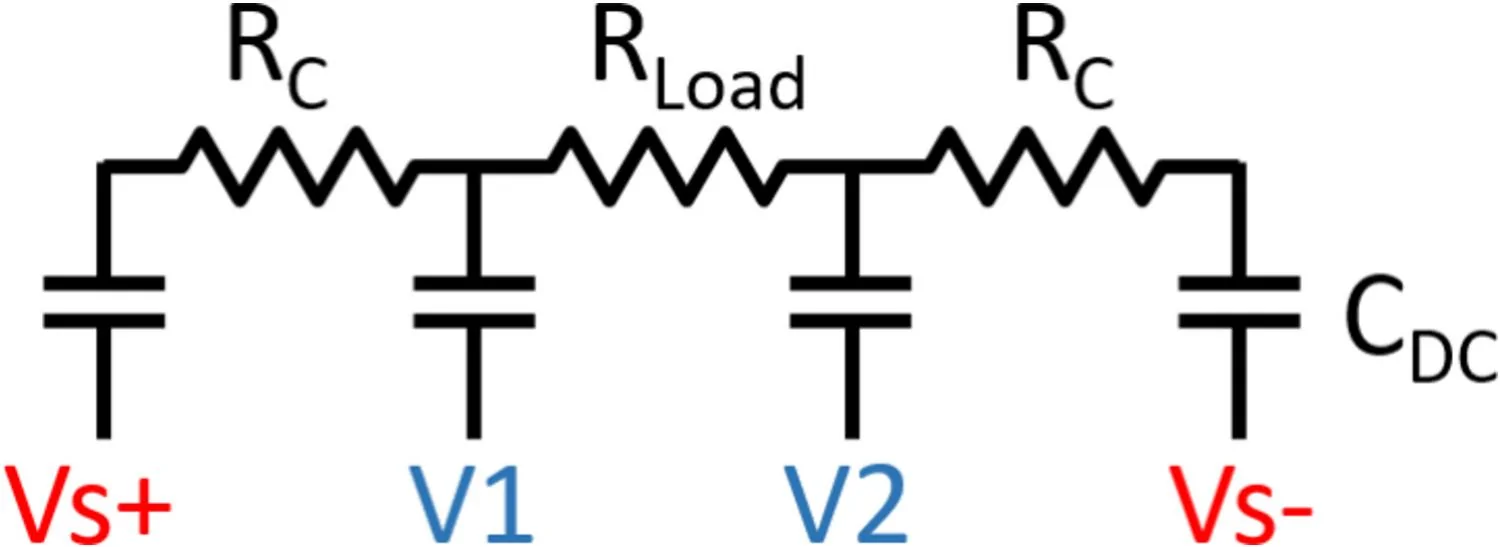
Evaluation circuit modeling a tetrapolar electrode configuration. Contact resistors (R_c_) model the electrode impedance on the voltage source pair (Vs+/Vs-), and the test load (R_Load_) is measured across the voltage pickup electrodes (V1/V2).

**Fig. 20:**
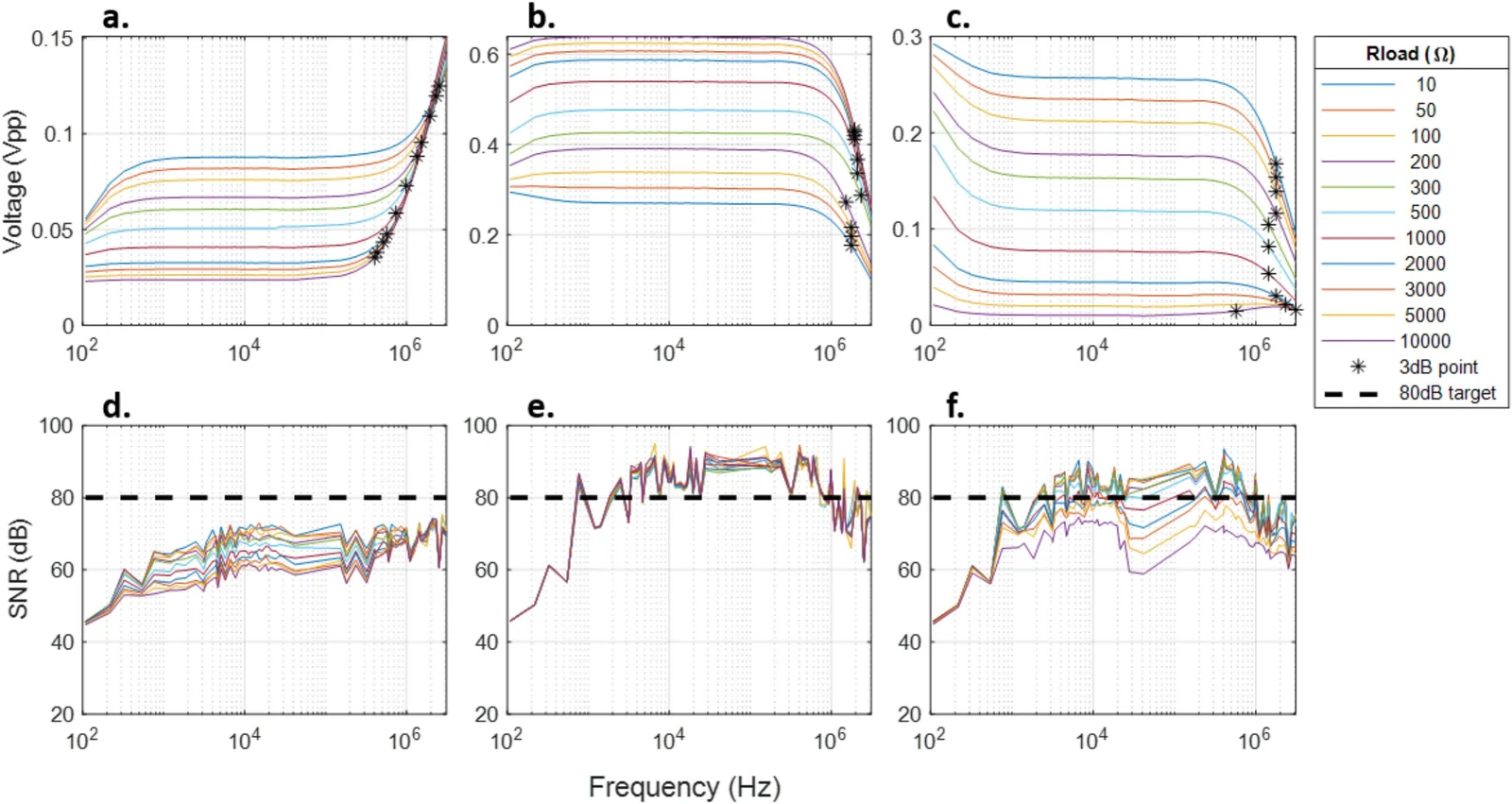
Frequency responses of DAQ voltages (a–c) and SNR (d-f) for loads 10 Ω to 10 kΩ over frequencies 100 Hz to 3 MHz. (a) The current sense (Visense) meets the bandwidth for small loads (<1 kΩ), with the 3 dB cutoff frequency reducing to 400 kHz for a 10 kΩ load. (b) Voltage pickup (Vpickup) channels 1 and (c) 2 meet the 100 Hz to 1 MHz target bandwidth for loads < 10 kΩ. SNR was computed over 100 measurements with 4 averages per measurement. (d) Visense achieves 60–70 dB due to its small amplitude (<100 mV) – up to 32 averages are required to achieve an SNR over 80 dB. The 80 dB SNR target is met by both Vpickup channels (e, f) for frequencies 1 kHz to 1 MHz.

The DAQ was evaluated using a tetrapolar configuration (Fig. 19), with 100 Ω contact resistors (R_c_) modeling electrode impedance. The frequency responses and SNR of the measured voltages are shown in Fig. 20. The voltage pickup (Vpickup channels 1 and 2) signals have flat responses over the 100 Hz to 1 MHz range for loads <10 kΩ, but the high-frequency 3 dB point on the I_sense_ signal (Visense) is reduced for loads >1 kΩ (400 kHz for a 10 kΩ load) due to bandwidth and slew rate limitations of the instrumentation amplifier used on the circuit. With the I_sense_ signal integrity being the limitation in computing load impedance, the full target bandwidth is only achievable for loads under 1 kΩ without calibration, which is sufficient for most imaging applications. Additionally, the I_sense_ voltage is less than 100 mV, and therefore requires far more averaging (up to 32 averages per measurement) to achieve the 80–90 dB SNR of the 200–600 mV signals on Vpickup Channel 1.

**Fig. 21:**
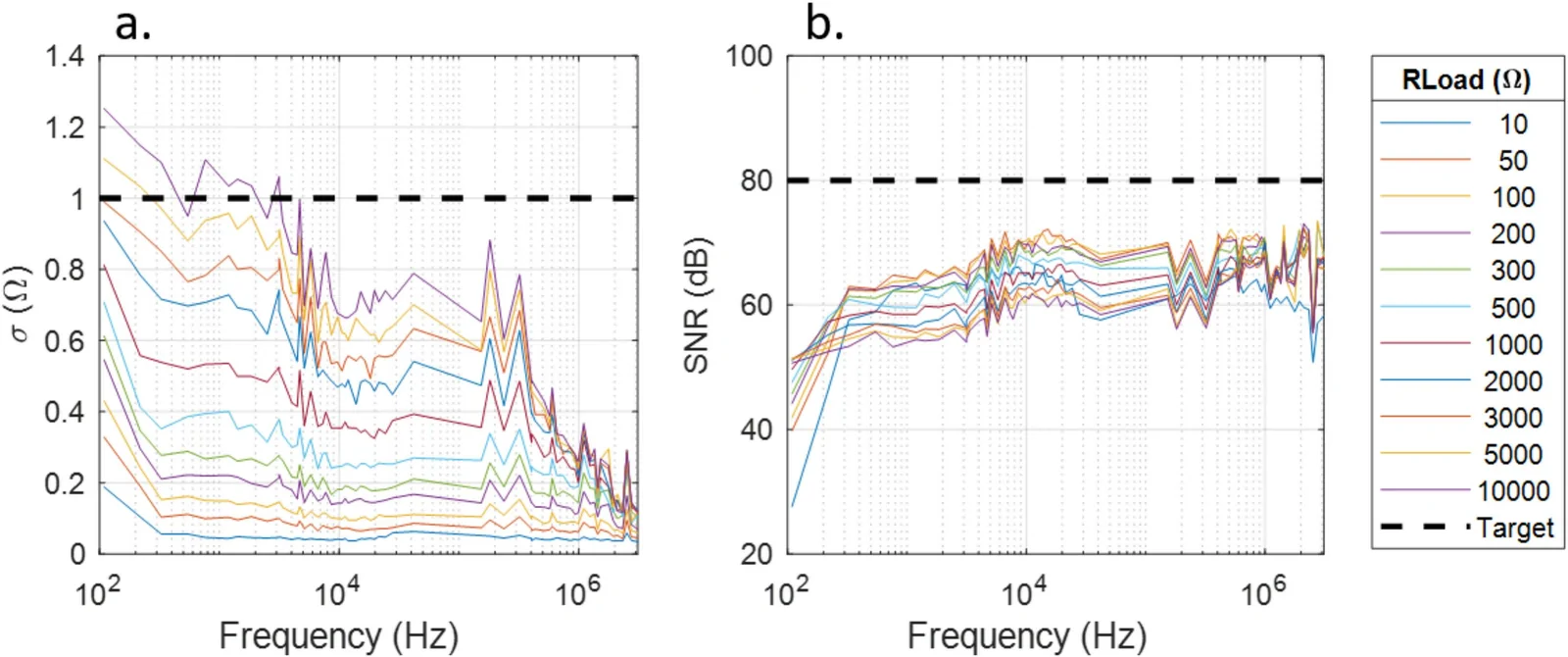
Frequency response of impedance precision (a) and SNR (b) using discrete loads 10 Ω to 10 kΩ over frequencies 100 Hz to 3 MHz. Precision (σ) was computed over N = 100 measurements with 4 averages per measurement, where measured impedance (Z) = Vload/Isense. a) Impedance precision is < 1 Ω for loads ≤ 3kΩ, with a maximum of 1.2 at 100 Hz with a 10 kΩ load. b) Impedance SNR is limited by the small Isense signal, which has an SNR of 60–70 dB with this configuration.

The load impedance was calculated from the tetrapolar measurements using Ohm's Law, R_load_ = V_load_/I_sense_, where V_load_ = V1-V2 (Fig. 19). Impedance precision (σ) and SNR were evaluated, where σ was computed as the standard deviation of 100 repeated measurements using MATLAB’s std function, and SNR was calculated with (6). As shown in Fig. 21, the impedance precision is better than 1 Ω for loads ≤ 3 kΩ, with a maximum of 1.2 Ω for the 10 kΩ load. The measured SNR ranges from 60-70 dB for loads ≤ 3 kΩ and is limited by the small I_sense_ voltages (<100 mV). For applications with large loads (>1 kΩ), the SNR can be improved by increasing the amplitude of the voltage source by removing the digital division factor (refer to *Section 2.2.2. Voltage Source and Current Sense* for details). The default division factor of 16 is included to ensure the injected current remains below the IEC 60601-1 limit for small loads (<1 kΩ), but this factor contributes to a decrease in SNR of ∼24 dB; for higher impedance loads, it can be decreased or removed while still maintaining compliance with the safe current limit.

The frequency-dependent trends shown in Fig. 20, Fig. 21 arise from several interacting effects within the measurement data path. At low frequencies, the impedance of the DC-blocking capacitors and the modeled electrode interface contribute additional series impedance, increasing measurement error for high-impedance loads. At higher frequencies, the finite bandwidth of the AFE, along with the MUX source/drain capacitance and PCB parasitics, introduces attenuation and phase shift that reduce the usable bandwidth, particularly for loads above 1 kΩ. The voltage-source architecture also exhibits load-dependent amplitude variation due to the finite output impedance of the source path, while the current-sense instrumentation amplifier ultimately limits the high-frequency performance of the I_sense_ measurement. Consequently, the measured impedance precision, bandwidth, and SNR represent the combined effects of these frequency-dependent error sources under the operating conditions evaluated.

### 8.2 EIT imaging results

**Fig. 22:**
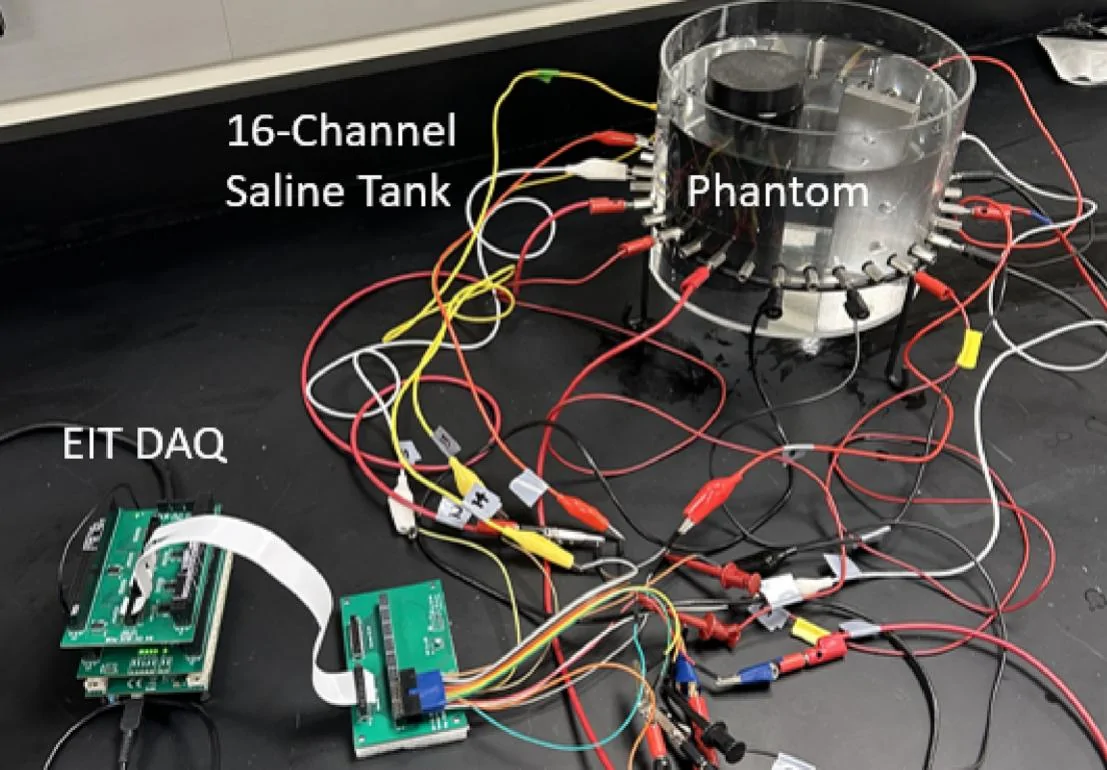
EIT imaging setup using the DAQ and 16-channel cylindrical tank.

To assess imaging capabilities, the EIT DAQ was used to acquire impedance data on a 16-electrode cylindrical phantom, as shown in Fig. 22. While the benchtop hardware characterization evaluated all 64 DAQ channels, imaging experiments were performed using a 16-electrode phantom because a 64-electrode phantom was not available. Inclusions were placed in varying locations within the tank to evaluate localization accuracy and ability to resolve both high- and low-contrast inclusions, and time-difference EIT images were reconstructed using saline as the reference dataset. 2D difference EIT images were produced using a one-step Gauss-Newton algorithm with Laplace smoothing regularization and a dual mesh approach [30] using a custom MATLAB toolbox [31]. The dual mesh maps the 3D Jacobian information, computed using the lead-field approach [32] and the complete electrode model [33], onto a 2D 30x30 uniform grid of voxels. The Tikhonov factor was heuristically selected.

**Fig. 23:**
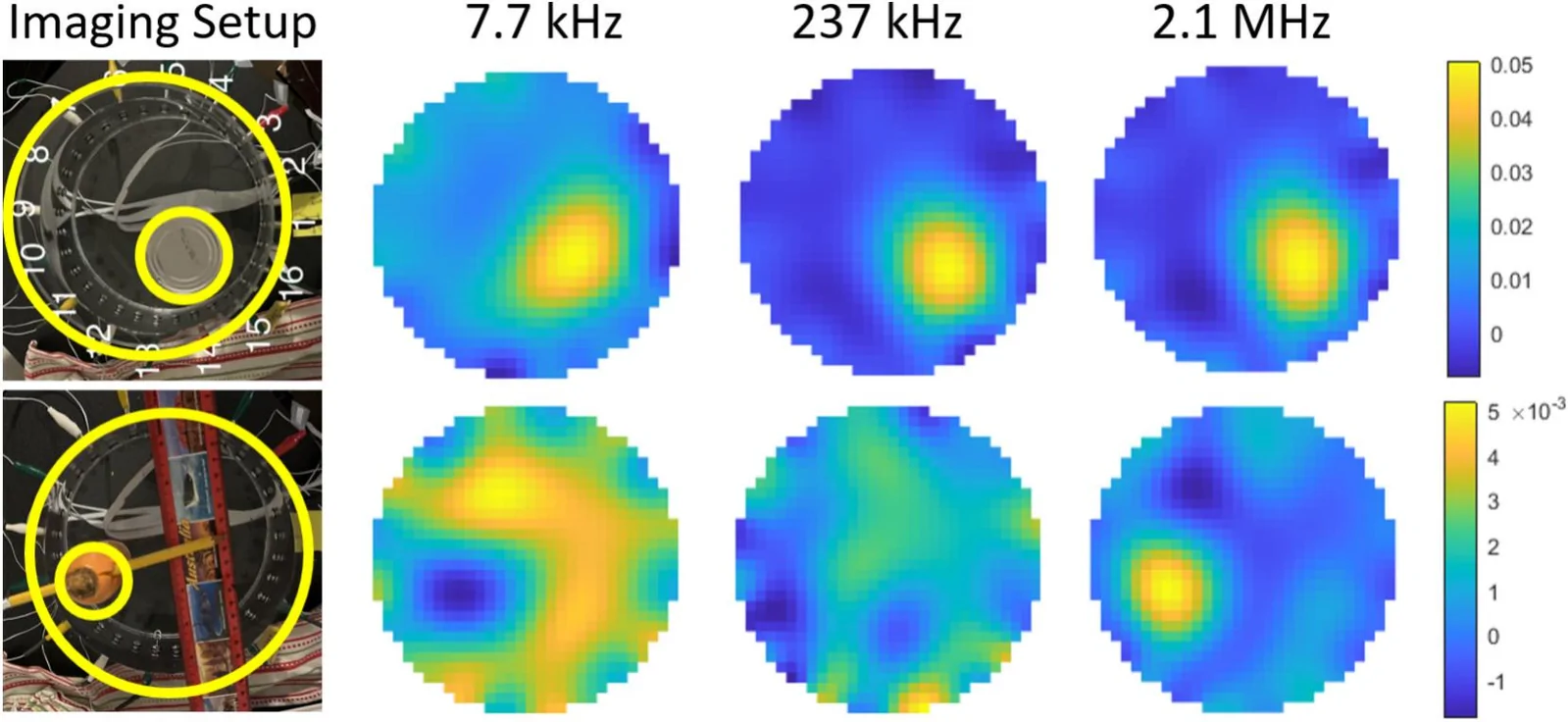
EIT phantom imaging results of a metal can (top row) and carrot (bottom row) in saline. Metal is highly conductive with no dispersion in this frequency range: the high-conductivity region (yellow) in the EIT image correctly corresponds to the inclusion location at all three frequencies shown. The carrot is biological with visible dispersion in this frequency range: the inclusion appears as a low conductivity region (blue) in the 7.7 kHz image, disappears in the 237 kHz image (i.e., is a similar conductivity as the background saline), and reappears in the 2.1 MHz image as a high conductivity inclusion. (For interpretation of the references to colour in this figure legend, the reader is referred to the web version of this article.)

The high-contrast metal inclusion (cylindrical can) is clearly visible in the EIT reconstructions in Fig. 23, where the yellow region corresponds to the high-conductivity inclusion location. EIT reconstructions of a carrot suspended in saline show the ability to localize low-contrast inclusions over multiple frequencies as well. Interestingly, the carrot images appear to have a reversal of relative conductivity around 200 kHz due to the β-dispersion phenomenon of biological materials, versus the relatively stable images of the metal phantom, which in practice has a conductivity near infinity for all frequencies within the DAQ’s range.

**Fig. 24:**
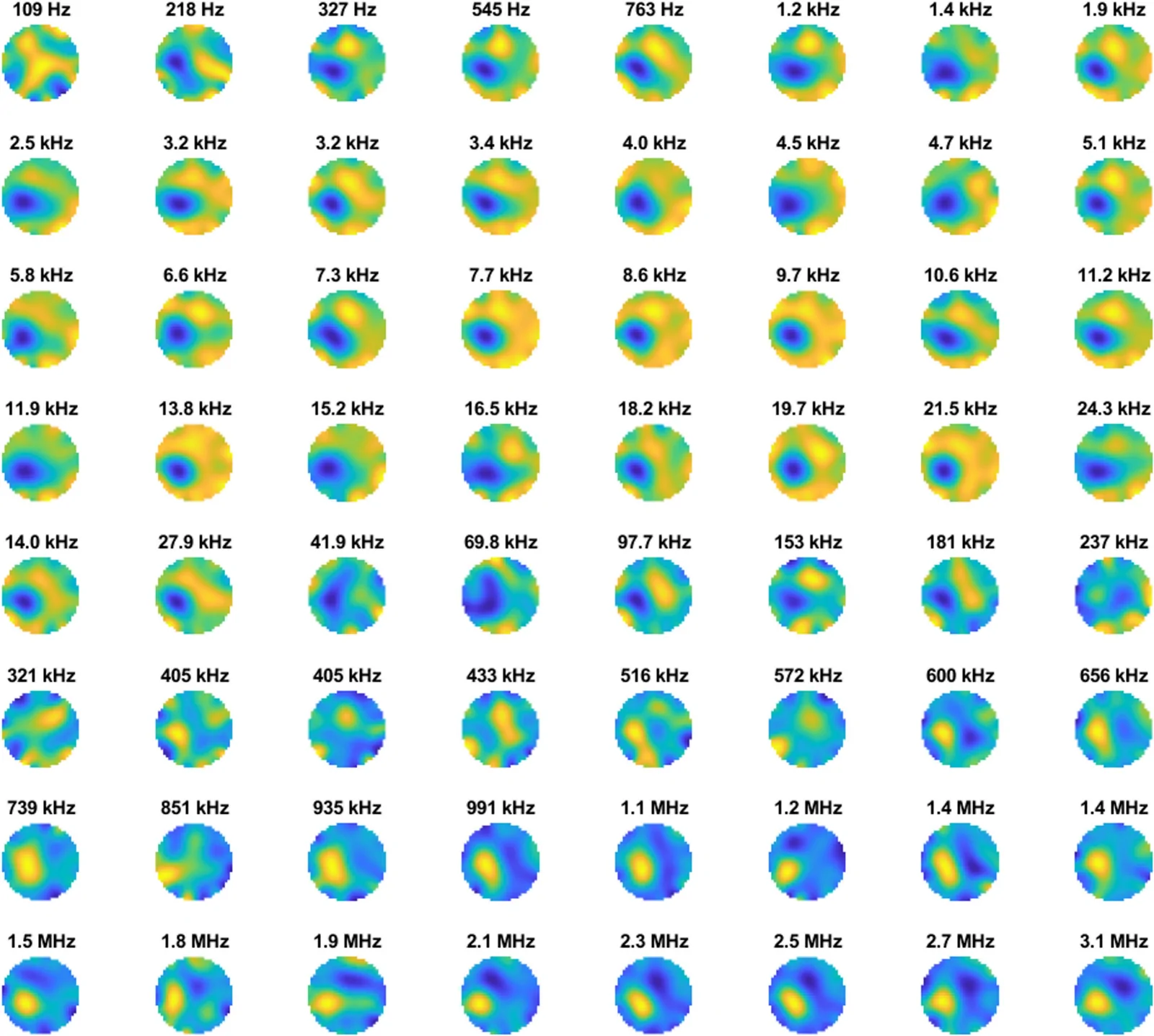
Multi-frequency EIT reconstructions of a carrot in saline show the ability to localize the inclusion over the full bandwidth. In the lower frequency images, the blue region (low conductivity) corresponds to the inclusion location. At high frequencies, the yellow region (high conductivity) corresponds to the inclusion location, indicating the β dispersion that occurs with biological materials over this frequency range. (For interpretation of the references to colour in this figure legend, the reader is referred to the web version of this article.)

To highlight multi-frequency DAQ capabilities for low contrast imaging, EIT reconstructions of the carrot at 64 frequencies ranging from 109 Hz to 3.1 MHz are shown in Fig. 24. The biological frequency dependence of the carrot inclusion is particularly apparent, as the pixels corresponding to the carrot location change from low to high conductivity (blue and yellow, respectively) relative to the background saline as the frequency increases.

**Fig. 25:**
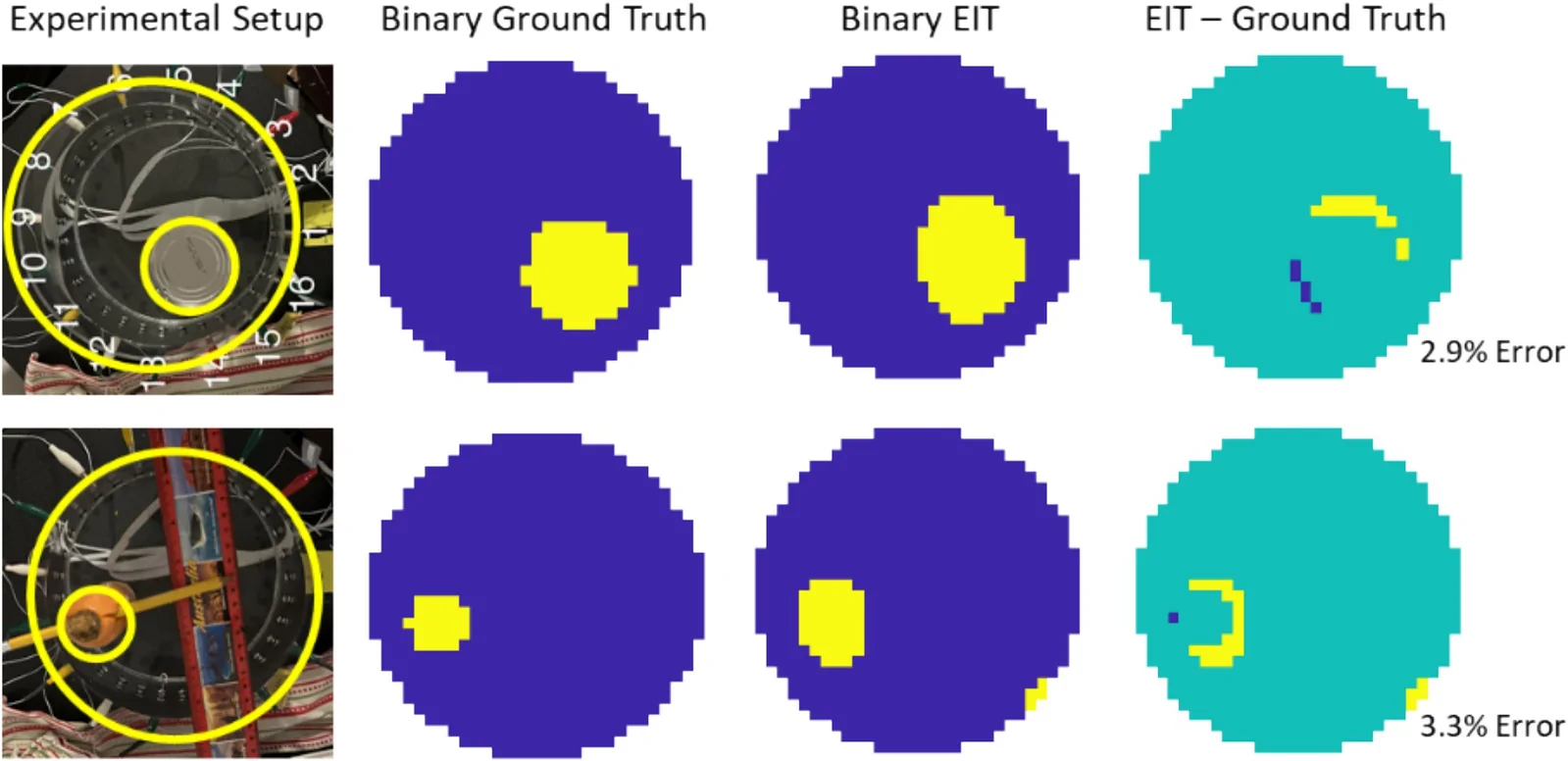
Localization error from phantom imaging results. Binary (threshold) EIT images show the ability to localize the phantoms with < 5% error.

To assess localization performance, pixel error was computed for both phantom configurations by comparing thresholded EIT images with their corresponding ground truth binary images. Pixel error was calculated by thresholding the reconstructed EIT image, subtracting the ground truth binary map, and computing the percentage of mismatched pixels. Because this metric depends on the selected image contrast and threshold values, it should be interpreted as an approximate measure of localization accuracy rather than an absolute performance metric. Fig. 25 shows that the pixel error is less than 5%, indicating successful localization of the inclusion. Further improvements in image resolution can be achieved through optimized source/pickup electrode patterns, advanced image reconstruction algorithms (e.g., GREIT), frequency-difference imaging, and digital filtering. The system’s 64-channel architecture also provides the flexibility to support higher-density electrode arrays than those evaluated in this work, offering a pathway toward improved spatial resolution in future studies. The high-precision impedance measurements produced by the DAQ are compatible with a wide range of EIT reconstruction techniques and can support future algorithmic developments, including data-driven approaches.

## 9 Conclusion

The FPGA-based EIT DAQ described here provides a novel set of attributes, in terms of compact size, high channel count, wide operating bandwidth, configurable acquisition modes, and open-source design, complementing existing EIT systems with different design priorities (Table 4, which includes prior hardware noted and the following systems [16], [19], [34], [35], [36]). As shown in Table 4, no single system outperforms the others across all performance metrics. Instead, the proposed system emphasizes portability, design flexibility, and multi-frequency acquisition while maintaining competitive imaging performance. The versatile acquisition scheme allows for high-speed multi-frequency imaging, which can reduce the effects of transient noise on EIT reconstructions and enable multi-frequency EIT of high-speed biological processes (e.g., cardiac output, pulmonary function). The wide bandwidth (100 Hz to 1 MHz) and high channel count (64) provide capabilities for imaging a wide range of pathologies, as the highest contrast frequency range is dependent on tissue type, and the number of electrodes required for sufficient imaging sensitivity depends on the clinical application. The performance achieved with this small form-factor system represents an important step towards portable, multi-application, real-time EIT imaging. In addition to ground-based biomedical applications, the modular FPGA architecture and compact, low-power design provide a foundation for future adaptation to resource-constrained environments, including aerospace platforms. While the prototype presented here is implemented using a commercial Artix-7 FPGA, the design is portable to radiation-tolerant or radiation-hardened FPGA families, with appropriate radiation-mitigation techniques required for future flight implementations.

**Table 4:** Comparison of EIT systems.

System	mfEIT (Yang, Jia)	KHU Mark2.5	Dartmouth (Rao)	Dartmouth (Khan)	Sciospec	RPI ACT-4	This system
Publication	Yang and Jia	Sohal et al & Wi et al	Rao et al	Khan et al	Sciospec 2024	Saulnier et al	2024
Year	2017	2014	2020	2014	Commercial	2007	2023
Small form factor	**Yes**	**Yes**	**Yes**	No	No	No	**Yes**
Open-source	**Yes**	No	No	No	No	No	**Yes**
# Channels	32	16	16	32	**16**–**128**	**72**	**64**
Bandwidth (Hz)	10 k–1 M	50–500 k	1 k–1 M	**100–10 M**	100–100 k	3 k–1 M	**100–1 M**
SNR (dB)	45.5–82.8	**80**	66–76	**>90**	N/A	**>90**	**>80 with averaging**
Max frame rate (fps)^1^	**546–1014**	**100**	<26	**110**	**100**	2.6	**>30**
Clinical application	3D cell culture	Long term monitoring	Prostate cancer	Prostate cancer	**Flexible**	Breast cancer	**Flexible**

^1^Frame-rate values are reported as provided in the referenced publications. Because acquisition protocols differ substantially between systems, these values are not directly comparable.

## CRediT authorship contribution statement

**Kendall R. Farnham:** Conceptualization, Formal analysis, Funding acquisition, Investigation, Methodology, Resources, Software, Validation, Visualization, Writing – original draft. **Ethan K. Murphy:** Writing – review & editing, Software. **Ryan J. Halter:** Writing – review & editing, Supervision, Funding acquisition.

## Declaration of competing interest

The authors declare that they have no known competing financial interests or personal relationships that could have appeared to influence the work reported in this paper.
